# Rational enhancement of the energy barrier of bis(tetrapyrrole) dysprosium SMMs *via* replacing atom of porphyrin core†
†Electronic supplementary information (ESI) available: Synthesis procedures of HOTBPP and HSTBPP; electronic absorption spectra of **1–3** in CHCl_3_; molecular packing of **1** and **2** in single crystals; temperature dependence of *χ*_m_ *T*, *M vs. H*/*T*, in-phase (*χ*′) and out-of-phase (*χ*′′) ac susceptibility curves of **1–3**; hysteresis loop of **1** and **2**; orientation of the local main magnetic axes of the ground Kramers doublet on Dy^III^ ions of **3**; analytical and mass spectroscopic data for the mixed double-deckers **1–3**; calculated energy levels (cm^–1^) and *m*_J_ values of the lowest Kramers doublets (KDs) of complexes **1–3**; calculated charge distribution of the coordinated atoms in **1–3**; crystallographic data for the mixed double-deckers **1** and **2** (CIF). CCDC 1058212 and 1058213. For ESI and crystallographic data in CIF or other electronic format see DOI: 10.1039/c5sc02314a



**DOI:** 10.1039/c5sc02314a

**Published:** 2015-07-20

**Authors:** Wei Cao, Chen Gao, Yi-Quan Zhang, Dongdong Qi, Tao Liu, Kang Wang, Chunying Duan, Song Gao, Jianzhuang Jiang

**Affiliations:** a Beijing Key Laboratory for Science and Application of Functional Molecular and Crystalline Materials , Department of Chemistry , University of Science and Technology Beijing , Beijing , 100083 , China . Email: jianzhuang@ustb.edu.cn ; Fax: +86-10-6233-2592; b State Key Laboratory of Fine Chemicals , Dalian University of Technology , Dalian , 116012 , China . Email: liutao@dlut.edu.cn; c Beijing National Laboratory of Molecular Science State Key Laboratory of Rare Earth Materials Chemistry and Applications , College of Chemistry and Molecular Engineering , Peking University , Beijing , 100871 , China . Email: gaosong@pku.edu.cn; d Jiangsu Key Laboratory for NSLSCS , School of Physical Science and Technology , Nanjing Normal University , Nanjing 210023 , China

## Abstract

With the coordination geometry of Dy^III^ being relatively fixed, oxygen and sulfur atoms were used to replace one porphyrin pyrrole nitrogen atom of sandwich complex [(Bu)_4_N][Dy^III^(Pc)(TBPP)] [Pc = dianion of phthalocyanine, TBPP = 5,10,15,20-tetrakis[(4-*tert*-butyl)phenyl]porphyrin]. The energy barrier of the compounds was enhanced three times, with the order of Dy^III^(Pc)(STBPP) > Dy^III^(Pc)(OTBPP) > [(Bu)_4_N][Dy^III^(Pc)(TBPP)] [STBPP = monoanion of 5,10,15,20-(4-*tert*-butyl)phenyl-21-thiaporphyrin, OTBPP = monoanion of 5,10,15,20-(4-*tert*-butyl)phenyl-21-oxaporphyrin]. Theoretical calculations offer reasonable explanations of such a significant enhancement. The energy barrier of 194 K for Dy^III^(Pc)(STBPP) represents the highest one among all the bis(tetrapyrrole) dysprosium SMMs, providing a strategy to rationally enhance the anisotropy and energy barrier *via* atom replacement.

## Abstract 2

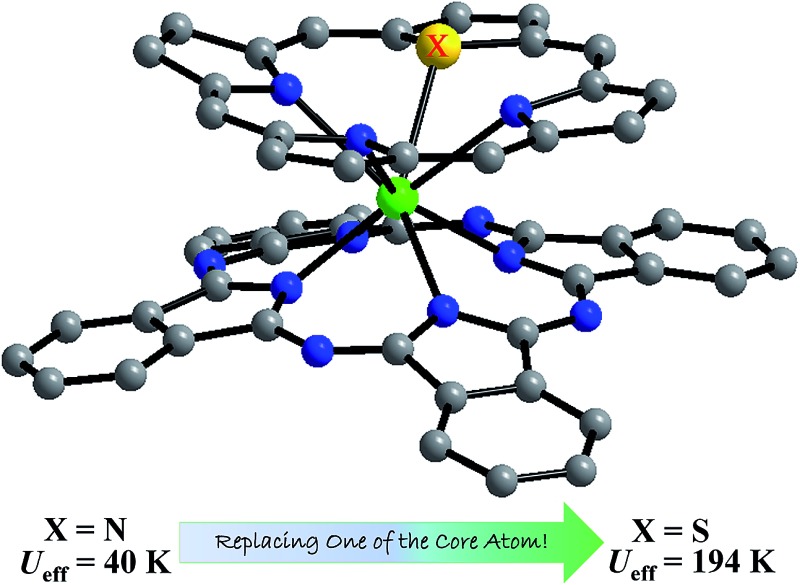
Replacing a porphyrin N atom induces higher electrostatic environment anisotropy around the Dy center, giving the highest energy barrier among bis(tetrapyrrole) Dy SMMs.

## Introduction

Single-molecule magnets (SMMs) are attracting considerable research interest because they exhibit slow relaxation, quantum tunneling of the magnetization and magnetic bistability, possessing potential applications in information storage, quantum computing and molecular spintronics.^1–3^ The magnetic bistability is essentially controlled by an energy barrier to spin reversal. The value of the energy barrier effectively defines the temperature range over which the magnetization of a SMM is blocked. Although various SMMs have been synthesized, it still remains a challenge to enhance the energy barrier and blocking temperature. Efforts have been made along two directions to increase the energy barrier. One is to develop molecules with large ground states, and the other is to maximize the anisotropy of the system.^2*a*^ Great progress has been made in increasing the spin ground state by controlling the intramolecular magnetic interactions to be ferromagnetic, and a maximum spin ground state of *S* = 45 has been obtained for a Fe_42_ molecule.^4^ Recently, a combination of theoretical and experimental studies have clearly revealed the importance of the electrostatic environment around the metal center in determining the anisotropy of the SMMs.^3f,5^ Theoretical studies have also rationalized the promotion of Dy^III^-based SMM behaviour through combining charged ligands together with neutral ones.^6^ However, it is very difficult to rationally enhance the anisotropy, because the anisotropy is extremely sensitive to small changes in the structures of complexes, as evidenced by the strong influence of the rotation of the hydrogen atoms in the coordinated water ligands in mononuclear Dy^III^ complexes on their SMM behavior.^3*e*^ We focused on multiple(tetrapyrrole) lanthanide multiple-decker complexes (tetrapyrrole = porphyrin and/or phthalocyanine), especially bis(tetrapyrrole) lanthanide double-decker SMMs, to increase the anisotropy *via* tuning the electrostatic environment.^7–12^ However, the reported examples are limited to modifying the tetrapyrrole peripheral substituents to tune the magnetic properties for bis(tetrapyrrole) lanthanide SMMs, which induces a relatively small effect on the magnetic properties, due to the weak influence on the electrostatic environment around metal centers.^10^ It is supposed that the coordination environments and electronic environments of lanthanide metal centers could be significantly tuned by replacing one or several porphyrin pyrrole nitrogen atom(s) with furan oxygen/thiophene sulfur atom(s) due to their different electronegativity, which will change the charge distribution and enhance the magnetic anisotropy and energy barrier.

In the present paper, core-modified porphyrin has been incorporated into bis(tetrapyrrole) lanthanide compounds for the first time, leading to unprecedented mixed (phthalocyaninato)(core-modified porphyrinato) dysprosium(iii) double-decker complexes Dy^III^(Pc)(XTBPP) [Pc = dianion of unsubstituted phthalocyanine, OTBPP = monoanion of 5,10,15,20-(4-*tert*-butyl)phenyl-21-oxaporphyrin, STBPP = monoanion of 5,10,15,20-(4-*tert*-butyl)phenyl-21-thiaporphyrin] [X = O (**1**), S (**2**)]. For the purpose of comparative study, [(Bu)_4_N][Dy^III^(Pc)(TBPP)] {TBPP = 5,10,15,20-tetrakis[(4-*tert*-butyl)phenyl]porphyrin} (**3**) was also prepared. Slow magnetic relaxation behavior for all three double-deckers indicates their single-molecule magnet nature. Impressively, fine core modification *via* changing only one of the four porphyrin pyrrole nitrogen atoms to oxygen/sulfur in the double-decker molecule induces significant enhancement of the magnetic performance, in terms of both the significantly increased energy barrier of 136 K and 194 K for **1** and **2**, respectively, in comparison with their porphyrin-containing analogue **3**, 40 K, and the observation of magnetic hysteresis loops for the former two double-deckers due to the significant enhancement of anisotropy over the oxygen/sulfur atom on the basis of theoretical calculations. The present result opens a new way towards optimizing the magnetic performance of sandwich-type multiple(tetrapyrrole) lanthanide SMMs.

## Results and discussion

### Synthesis and spectroscopic characterization

**Scheme 1:**
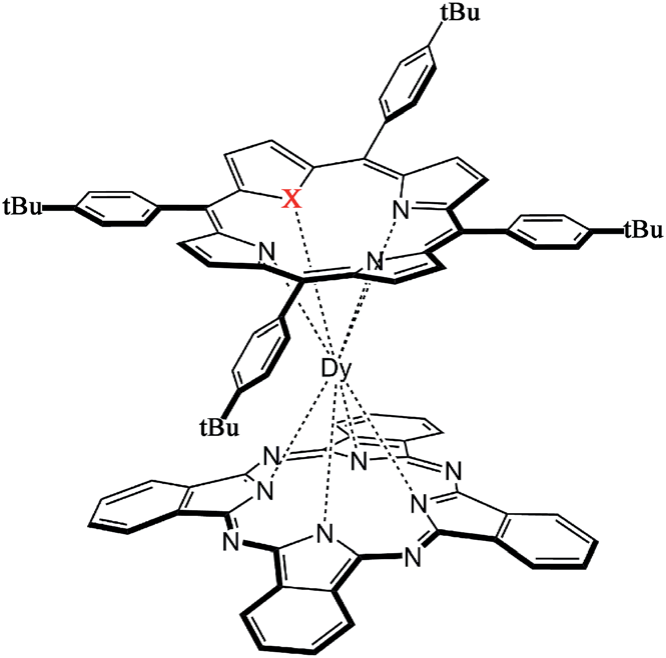
Schematic molecular structure of the sandwich-type mixed (phthalocyaninato)(core-modified porphyrinato) double-decker complexes **1** and **2** (X = O, S).

Since the synthesis of the first sandwich-type bis(tetrapyrrole) metal double-decker compound Sn(Pc)_2_ in 1936, significant progress has been made in this field through intensive studies of various kinds of multiple(tetrapyrrole) metal multiple-decker complexes (multiple = bis, tris, tetrakis, pentakis, hexakis; metal = rare earths, actinides, early transition and several main group metals) with a wide range of potential applications associated with their intriguing optical, electric and magnetic properties.^7–12^ However, core-modified porphyrins, interesting porphyrin isomers with the basic porphyrin framework preserved and at least one of the inner pyrrole nitrogen atoms replaced by another heteroatom, have never been incorporated into the sandwich complexes since they were first reported, in 1971.^13^ In the present paper, the core-modified porphyrins HOTBPP and HSTBPP [HOTBPP = 5,10,15,20-(4-*tert*-butyl)phenyl-21-oxaporphyrin, HSTBPP = 5,10,15,20-(4-*tert*-butyl)phenyl-21-thiaporphyrin] were prepared by a modified procedure of a published pathway^14^ (for details, please see the ESI†). Similar to their mixed (phthalocyaninato)(porphyrinato) rare earth double-decker analogues, core-modified porphyrin-containing mixed ring dysprosium double-decker complexes Dy^III^(Pc)(XTBPP) [X = O (**1**), S (**2**)], Scheme 1, were also synthesized by reaction of the half-sandwich compound Dy(Pc)(acac) (acac = acetylacetonate) with metal-free core-modified porphyrin. Treatment of Dy(Pc)(acac) with HXTBPP (X = O, S) in refluxing 1,2,4-trichlorobenzene (TCB) led to the isolation of the sandwich-type mixed (phthalocyaninato)(core-modified porphyrinato) dysprosium(iii) double-decker compounds Dy^III^(Pc)(XTBPP) [X = O (**1**), S (**2**)] in yields of 17 and 3.1%, respectively. Both compounds were soluble in common organic solvents such as CHCl_3_, CH_2_Cl_2_ and toluene, and could therefore be readily purified by column chromatography. For the purpose of comparative study, [(Bu)_4_N][Dy^III^(Pc)(TBPP)] (**3**) was also prepared according to a literature procedure.^7c,15^ The two newly prepared sandwich-type mixed (phthalocyaninato)(porphrinato) double-decker compounds **1** and **2** gave satisfactory elemental analysis data, as shown in Table S1 (ESI†). Their MALDI-TOF mass spectra clearly show intense signals for the corresponding protonated molecular ions [M + H]^+^, which are in good agreement with the corresponding calculated values (Table S1, ESI†).

The electronic absorption spectra of **1–3** were recorded in CHCl_3_, and the data are summarized in Table S2 (ESI†). As displayed in Fig. S1 (ESI†), these mixed ring double-decker compounds containing core-modified porphyrin gave strong phthalocyanine and porphyrin absorption bands at 334–346 and 412–472 nm, respectively, and several Q bands in the region of 482–834 nm. It is worth noting that the characteristic radical absorption band in the near-IR region for bis(tetrapyrrole) rare earth(iii) double-decker complexes containing an unpaired electron^9a,16^ was not observed in the electronic absorption spectra of both **1** and **2**, indicating their neutral nature with the composition of Dy^III^(Pc^2–^)(XTBPP^–^) [X = O (**1**), S (**2**)].

### Structural studies

**Fig. 1:**
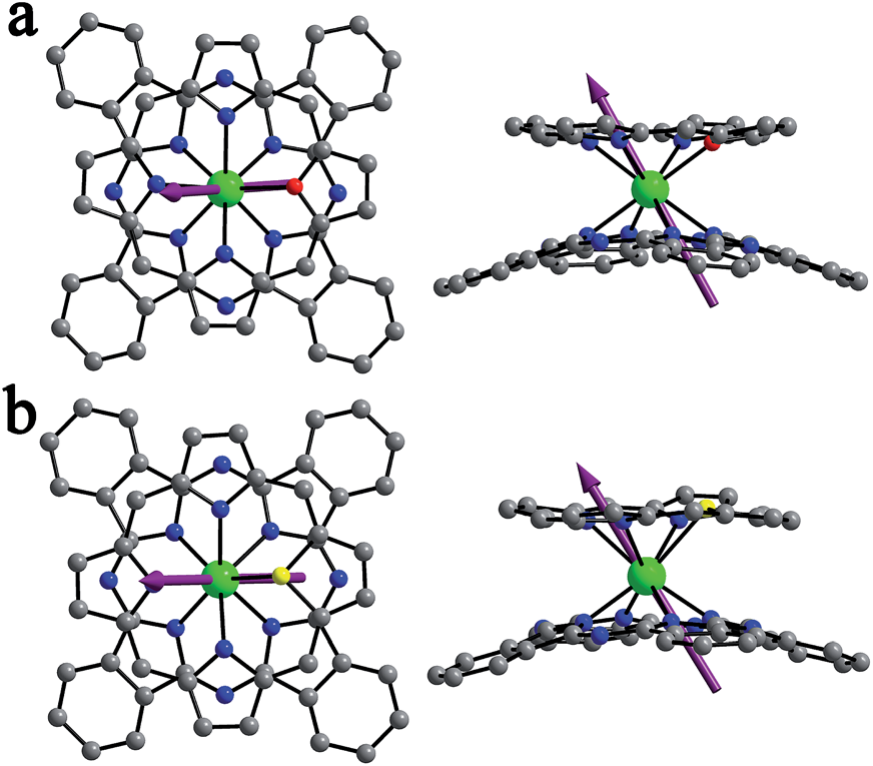
Molecular structures of Dy(Pc)(OTBPP) (**1**) and Dy(Pc)(STBPP) (**2**) in top and side views with the hydrogen atoms and (4-*tert*-butyl)phenyl groups omitted for clarity [Dy(iii) = green, C = grey, N = blue, O = red and S = yellow]. The purple arrows represent the easy axis calculated by using CASSCF calculations.

Single crystals of Dy^III^(Pc)(OTBPP) (**1**) and Dy^III^(Pc)(STBPP) (**2**) suitable for X-ray diffraction analysis were obtained by diffusing methanol into chloroform/toluene (1 : 3) and chloroform solutions, respectively, of the corresponding compounds. Despite the great effort paid, trials towards obtaining single crystals of [(Bu)_4_N][Dy^III^(Pc)(TBPP)] (**3**) still failed due to the easy oxidization of this compound in the reduced state into the neutral unprotonated state Dy^III^(Pc)(TBPP) in solution. The molecular structures of **1** and **2** were determined by X-ray diffraction analysis. As can be seen in Fig. 1, the central dysprosium(iii) ion in both **1** and **2** is eight-coordinated by the four isoindole nitrogen atoms of the phthalocyanine ligand together with three pyrrole nitrogen atoms and one oxygen/sulphur atom of the core-modified porphyrin ligand, resulting in an approximately square-antiprismatic coordination polyhedron for the dysprosium ion. The dysprosium metal center lies at 2.505–2.577 and 2.376–2.413 Å from the nitrogen atoms of the pyrrole ring and isoindole ring, respectively. Meanwhile, the bond lengths between Dy(iii) and the heteroatom are 2.510 and 2.769 Å for **1** and **2**, increasing along with the increase of the ionic radius from O to S. In comparison with the bond lengths of Dy–N(isoindole), the larger bond lengths of Dy–N(pyrrole) and Dy–X (X = O, S) indicate weaker bonding between Dy(iii) and the core-modified porphyrin ligand. The twist angle *φ*, which is defined as the rotation angle of one coordination square away from the eclipsed conformation of the twos, amounts to 44.22 and 43.30° for **1** and **2**, respectively, both of which slightly deviate from the ideal square-antiprismatic symmetry (*φ* = 45°). These results suggest that the influence of the core-modified porphyrin ligand on the square-antiprismatic coordination geometry of the center dysprosium ion in the mixed ring double-deckers is little in terms of the twist angle.^7*b*^

The crystal packing diagrams of the mixed ring double-deckers **1** and **2** are shown in Fig. S2 and S3 (ESI†). The dysprosium double-decker molecules in the single crystal of **1** are well separated by the chloroform and toluene molecules, with the nearest Dy···Dy distance being 12.481 Å. However, the adjacent double-decker molecules in the single crystal of **2** form a dimer *via* the π–π interaction of phthalocyanine ligands between neighboring double-decker molecules, resulting in a shorter intermolecular Dy···Dy distance than **1**, of 9.311 Å. This, however, appears to be still large enough to avoid obvious intermolecular magnetic interactions, according to previous reports.^17^ More detailed crystallographic and structural data for these compounds are summarized in Tables S3 and S4 (ESI†).

### Magnetic properties

The temperature dependence of the magnetic susceptibility data for **1–3** was collected in the temperature range of 2–300 K under an applied field of 2 kOe. As can be seen in Fig. S4–S6 (ESI†), the curves of the magnetic susceptibility (*χ*_M_ *T*) for **1–3** show their temperature-dependent character. The *χ*_M_ *T* values of 14.11, 14.13 and 14.12 cm^3^ K mol^–1^ for **1–3** at 300 K are consistent with the theoretical value of 14.17 cm^3^ K mol^–1^ expected for a free Dy(iii) ion [^6^H_15/2_, *S* = 5/2, *L* = 5, *g* = 4/3].^18^ When the temperature was lowered, the *χ*_M_ *T* values of these three compounds decreased slowly until about 100 K, then to minimum values of 11.16, 10.59 and 10.09 cm^3^ K mol^–1^ for **1–3** at 2 K, respectively, due to the depopulation of excited Stark sublevels.^17,18^ In addition, as shown in Fig. S7–S12 (ESI†), the maximum value of *M*(*H*) for **1–3** at 2 K and 50 kOe ranges from 5.54 to 6.06*Nβ*, which considerably deviates from the theoretical magnetization saturation value of 10*Nβ*.^7b,17,18^ As can also be seen in these figures, the non-superposition field-dependence magnetization curves obtained at 2.0, 3.0 and 5.0 K for **1–3** indicate significant magnetic anisotropy for the dysprosium ion in these double-decker complexes.^19^

The dynamic magnetic properties were studied on multicrystalline powder samples of **1–3** in a 3.0 Oe ac field oscillating at 10–997 Hz. Fig. S13 and S14 (ESI†) show the plots of *χ*′ *vs. T* and *χ*′′ *vs. T* in a zero dc magnetic field for these compounds. As can be seen, all these double-deckers exhibit a frequency-dependent character in the in-phase signal (*χ*′) and out-of-phase signal (*χ*′′), indicating the slow relaxation of magnetization and revealing their SMM nature. Interestingly, frequency-dependent character was observed for both **1** and **2** in a much wider temperature range than that for **3**. Unfortunately, further understanding of the corresponding relaxation process for these three compounds failed, since *χ*′′ peaks were only observed at 997 Hz due to the fast relaxation associated with the quantum tunneling of magnetization (QTM) at zero dc magnetic field.^7b,17,18^

**Fig. 2:**
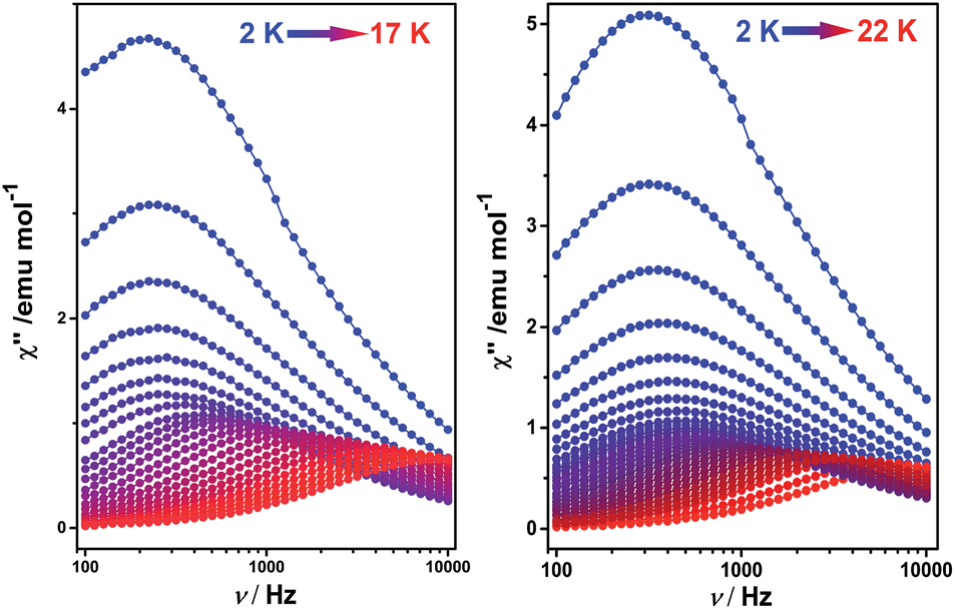
Frequency dependence of the out-of-phase (*χ*′′) ac susceptibility of **1** (left) and **2** (right), under zero applied dc field.

**Fig. 3:**
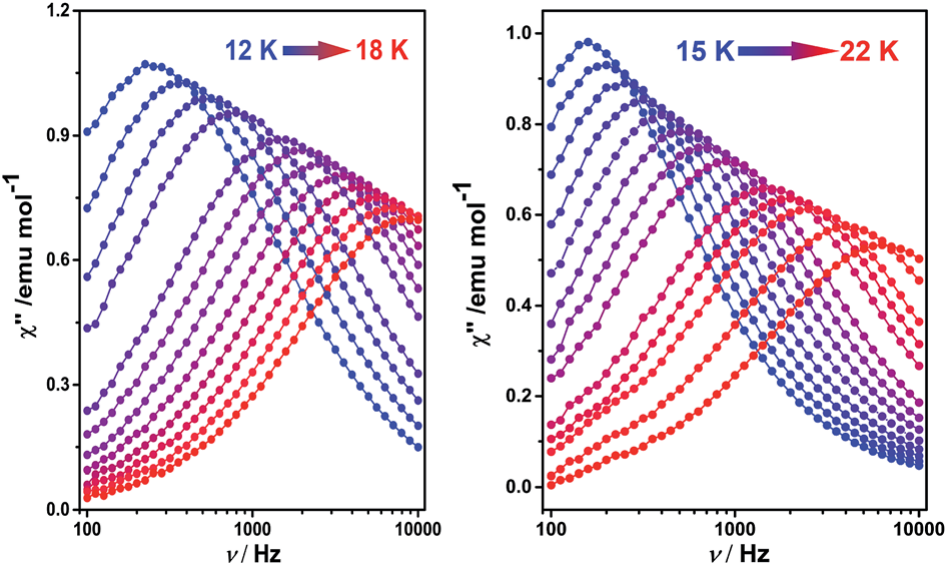
Frequency dependence of the out-of-phase (*χ*′′) ac susceptibility of **1** (left) and **2** (right), under a 2000 Oe applied dc field.

**Fig. 4:**
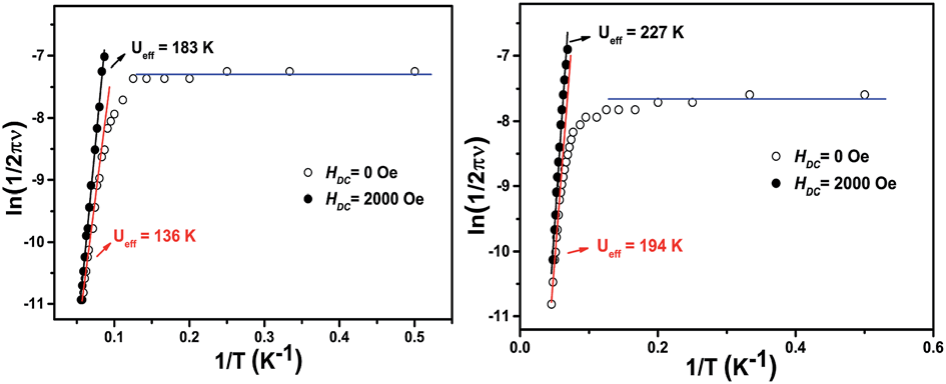
The plots of ln(*τ*) *vs.* 1/*T* for **1** (left) and **2** (right), under zero dc field and under a 2000 Oe dc field.

To better understand the magnetic behavior of these mono-dysprosium-containing double-decker compounds, frequency (*ν*) dependence of alternating current magnetic susceptibility measurements were performed in a 3.0 Oe ac field oscillating at 100–10 000 Hz. As shown in Fig. 2, well-resolved out-of-phase ac susceptibility maxima that vary with frequency can be found from 9 to 17 K for **1** and from 13 to 22 K for **2**, despite the observation of temperature-independent behavior associated with the QTM process in the low temperature region.^17*b*^ To restrain the quantum tunneling effect, a dc field was then applied. As shown in Fig. 3, S14 and S15 (ESI†), under an applied 2000 Oe magnetic field, the ac susceptibility data for **1–3** show an overall reduction in height due to saturation effects that depress the susceptibility,^20^ and the observation of the clear *χ*′′ peaks for **1–3** indicates an effective suppression of the QTM. On the basis of a thermally-activated mechanism, *τ* = *τ*_0_ exp(*U*_eff_/*kT*) and *τ* = 1/(2π*ν*), the Arrhenius law fitting for the data under zero dc magnetic field was carried out. As shown in Fig. 4, a linear relationship exists between ln(*τ*) and 1/*T* for both **1** and **2** in the high-temperature regime, which in turn results in a barrier *U*_eff_ = 136 K and *τ*_0_ = 8.0 × 10^–8^ s for **1** together with *U*_eff_ = 194 K and *τ*_0_ = 4.7 × 10^–8^ s for **2**. Impressively, such high energy barriers as those revealed for both **1** and **2** in the present case have never been reported for bis(tetrapyrrole) dysprosium SMMs, with the previous largest value of 79 K having been revealed for [Dy^III^{Pc(OC_2_H_5_)_8_}_2_]^+^(SbCl_6_).^8*i*^ As for the reference compound **3**, the energy barrier is only 40 K with *τ*_0_ = 9.4 × 10^–7^ under zero dc field, which is in line with those disclosed for other mixed (phthalocyaninato)(porphrinato) dysprosium double-decker analogues.^7*b*^ It is also worth pointing out that a much stronger QTM process was observed for **1** and **2** than for **3** based on ac susceptibility data (Fig. 2 and S16, ESI†), which can be attributed to the larger deviation from ideal *D*_4d_ symmetry after replacing one coordinated nitrogen atom with an oxygen/sulphur atom in the former two compounds.^3e,7b,7j^ This in turn results in the different tendencies of the ln(*τ*) *versus* 1/*T* plots, and should be responsible for the decreasing of the relaxation time in the low temperature region for **1** and **2** in comparison with **3**.

**Fig. 5:**
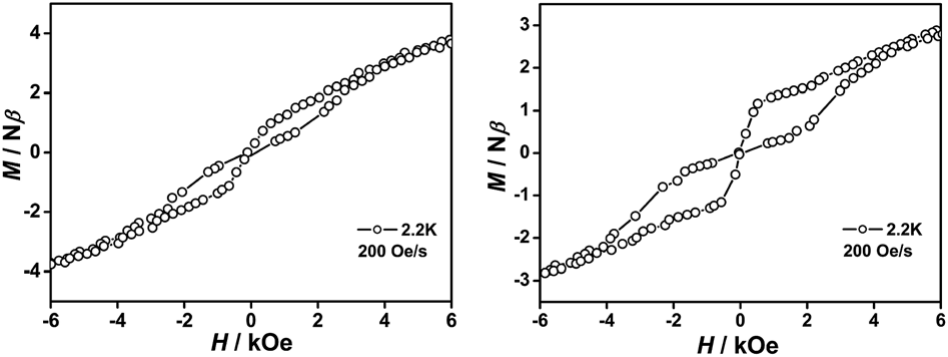
Hysteresis loops of **1** and **2** at 2.2 K with a 200 Oe s^–1^ sweep rate of the magnetic field.

In addition to their frequency-dependent and temperature-dependent characteristics, butterfly-shaped hysteresis loops emerged at 3 and 3.5 K, respectively, for **1** and **2** (Fig. 5, S18 and S19, ESI†), under a 200 Oe s^–1^ sweep rate of the magnetic field. As expected, the hysteresis loops for these two core-modified porphyrin-containing mixed ring double-decker complexes became larger as the temperature decreased. For the reference compound **3**, no hysteresis loop was revealed under the present measurement conditions. This actually represents the first observation of hysteresis loops for mixed (phthalocyaninato)(porphyrinato) lanthanide sandwich SMMs.

To further evaluate the magnetic properties of this series of compounds, complete-active-space self-consistent field (CASSCF) calculations have been carried out with the MOLCAS 7.8 program package.^21^ As can be seen in Table S6 (ESI†), compounds **1** and **2** show ground states with the highest *m*_J_ = ±15/2 component, while compound **3** shows a ground state with the second highest *m*_J_ = ±13/2 component, all of which behave with typical Ising ground states with an allowed transition to give rise to their SMM behavior. However, the calculated effective *g*_z_ values for **1** and **2** are 18.800 and 19.285, respectively, which approach closer to the Ising-limit value of 20, indicating stronger axial-anisotropy in comparison with that for **3**, where *g*_z_ = 17.270. Additionally, the energy barriers between the ground states and the first excited states of complexes **1–3** are determined to be 96.3, 120.5 and 26.3 cm^–1^, respectively, which are in good agreement with the experimental values fitted from the Arrhenius law. The easy axis orientation in **1–3** was also obtained from theoretical calculations (Fig. 1 and S20, ESI†). The angle between the easy axis of compound **3** and the N4 plane of the phthalocyanine ligand (defined by the four isoindole nitrogen atoms) is determined to be 87.2°, which changes drastically to 60.0° and 56.7° for **1** and **2**, respectively. For the purpose of rationalizing the magnetic behavior of these mono-dysprosium-containing double-decker SMMs, a Mulliken population analysis of relevant atom charges has been performed (Table S7, ESI†). The charges show an average distribution over the isoindole nitrogen atoms and pyrrole nitrogen atoms, ranging from –0.81 to –0.89e in all these three complexes. In good contrast, the negative charge on the oxygen atom in **1** is decreased significantly to –0.75e, which even changes to +0.15e on the sulfur atom in **2**. Nevertheless, the Dy–X [X = O (**1**), S (**2**)] bond lengths are longer than those of Dy–N(pyrrole) in **3**, according to the simulated structure result for this compound (Table S4, ESI†), indicating weaker bonding between Dy(iii) and the core-modified porphyrin ligand for **1** and **2** than that for **3**. On the basis of the experimental and calculation results, we can find that replacing only one of the four porphyrin pyrrole nitrogen atoms with oxygen/sulfur in the double-decker molecule leads to the decreased charge distribution and increased coordination bond length of the relevant atoms, and thus reduces the electrostatic repulsion between the ligands and f-electron charge clouds in the present case, which stabilizes the ground states with the largest *m*_J_ quantum number and facilitates axial anisotropy of dysprosium(iii) ions in **1** and **2** in comparison with those in **3**.^5*f*^ Actually, this result is also in line with the calculation result that a dysprosium ion located in a certain coordination geometry with a different charge distribution of ligands can promote magnetic anisotropy.^6^ Moreover, since the oxygen atom or sulfur atom lies approximately on the hard plane (perpendicular to the easy axis) of the corresponding compound, the decreased negative charge on the heteroatom and increased Dy–X [X = O (**1**), S (**2**)] bond lengths could result in a more anisotropic dysprosium ion, thus leading to the energy barrier of **2** being higher than that of **1**.^3f,22^

## Conclusions

Briefly summarizing the above, the first core-modified porphyrin-containing (phthalocyaninato)(core-modified porphyrinato) dysprosium(iii) sandwich complexes have been prepared and structurally characterized. Both double-decker compounds display significantly enhanced energy barriers, which have never been revealed among bis(tetrapyrrole) dysprosium-based SMMs, due to the significantly decreased charge distribution over the oxygen/sulfur atom (on the basis of the DFT calculations) in combination with the increased bond lengths between the dysprosium(iii) ion and the heteroatom. The present work seems to open a new way towards optimizing the magnetic performance of multiple(tetrapyrrole) lanthanide multiple-decker SMMs.

## Experimental section

### General

TCB and dichloromethane were freshly distilled from CaH_2_ under nitrogen. Column chromatography was carried out on silica gel columns (Merck, Kieselgel 60, 70–230 mesh) with the indicated eluents. The compounds Dy^III^(Pc)(TBPP),^15^ Dy(acac)_3_·*n*H_2_O^23^ and Dy(Pc)(acac)^24^ were prepared according to literature procedures. The core-modified porphyrins HOTBPP and HSTBPP, as well as their corresponding precursors, were also prepared according to a published procedure.^14^ Unless otherwise noted, all other reagents and solvents were used as received.

Electronic absorption spectra were recorded on a Lambda 750 spectrophotometer. MALDI-TOF mass spectra were taken on a Bruker BIFLEX III ultra-high resolution Fourier transform ion cyclotron resonance (FT-ICR) mass spectrometer, with α-cyano-4-hydroxycinnamic acid as the matrix. Elemental analyses were performed on an Elementar Vavio El III elemental analyzer. IR spectra were recorded in KBr pellets with 2 cm^–1^ resolution using a Bruker Tensor 37 spectrometer. Magnetic measurements were performed on a Quantum Design MPMS XL-5 SQUID magnetometer on polycrystalline samples. Data were corrected for the diamagnetism of the samples using Pascal’s constants, and that of the sample holder by measurement. Measurements of alternating current (ac) susceptibilities were carried out in the 100–10 000 Hz frequency range using a Quantum Design PPMS-9T. The measurements of hysteresis loops for compounds **1** and **2** were conducted on a PPMS-VSM.

#### Preparation of mixed (phthalocyaninato)(5,10,15,20-(4-*tert*-butyl)phenyl-21-oxaporphyrinato) dysprosium double-decker complex Dy^III^(Pc)(OTBPP) (**1**)

A mixture of 5,10,15,20-(4-*tert*-butyl)phenyl-21-oxaporphyrin (HOTBPP) (84.0 mg, 0.10 mmol) and Dy(Pc)(acac) (93.0 mg, 0.12 mmol) in TCB (4 mL) was heated to reflux under a nitrogen atmosphere for about 6 h. After a brief cooling, the solvent was removed under reduced pressure and the residue was chromatographed on a silica gel column with CH_2_Cl_2_ as the eluent. After the first green band containing bis(phthalocyaninato) dysprosium(iii) double-decker, a deep green band containing the target mixed (phthalocyaninato)(5,10,15,20-(4-*tert*-butyl)phenyl-21-oxaporphyrinato) dysprosium double-decker complex Dy^III^(Pc)(OTBPP) (**1**) was developed. The column was further eluted with triethylamine and CH_2_Cl_2_ (1 : 99 v/v), giving a brown band containing the unreacted metal-free HOTBPP. Repeated chromatography, followed by recrystallization from CHCl_3_ and MeOH, gave **1** as a dark green powder (24.0 mg, 17.0%).

#### Preparation of mixed (phthalocyaninato)(5,10,15,20-(4-*tert*-butyl)phenyl-21-thiaporphyrinato) dysprosium double-decker complex Dy^III^(Pc)(STBPP) (**2**)

By utilizing the above-mentioned reaction procedure for the preparation of Dy^III^(Pc)(OTBPP) (**1**) with 5,10,15,20-(4-*tert*-butyl)phenyl-21-thiaporphyrin (HSTBPP) (85.7 mg, 0.10 mmol) instead of HOTBPP, the target mixed ring double-decker compound Dy^III^(Pc)(STBPP) (**2**) was isolated, by chromatography on a silica gel column with CH_2_Cl_2_ as the eluent followed by recrystallization from CHCl_3_ and MeOH, as a dark green powder (4.7 mg, 3.1%).

#### Preparation of [(Bu)_4_N][Dy^III^(Pc)(TBPP)] (**3**)

Hydrazine hydrate (0.1 mmol dm^–3^) in CH_3_OH (10 mL) was added to a solution of Dy^III^(Pc)(TBPP) (30.0 mg, 0.02 mmol) in CH_2_Cl_2_ (100 mL) and stirred at room temperature under nitrogen for 1 h. Then an excess amount of tetra(*n*-butyl)ammonium hydroxide (50% in water) was added and the mixture was stirred for another 0.5 h. The dark green precipitate was isolated by filtration, washed with water and methanol and dried in a vacuum. The target compound was obtained as a dark green powder in a yield of 31.6 mg (90.0%).

### X-ray crystallographic analysis

Single crystals of Dy^III^(Pc)(OTBPP) (**1**) and Dy^III^(Pc)(STBPP) (**2**) suitable for X-ray diffraction analysis were grown by diffusing methanol into the chloroform/toluene (1 : 3) and chloroform solutions, respectively, of the corresponding compounds. Crystal data and details of the data collection and structure refinement are given in Tables S3 and S4 (ESI†). Data were collected on an Oxford Diffraction Gemini E system with Cu_Kα_ radiation (*λ* = 1.5418 Å) or Mo_Kα_ radiation (*λ* = 0.71073 Å) at 150 K, using an *ω* scan mode with an increment of 1°. Preliminary unit cell parameters were obtained from 30 frames. Final unit cell parameters were obtained by global refinements of reflections obtained from integration of all the frame data. The collected frames were integrated using the preliminary cell-orientation matrix. The software SMART was used for data collecting and processing, ABSpack was used for absorption correction^25^ and SHELXL was used for space group and structure determination, refinements, graphics and structure reporting.^26^ CCDC-; 1058212 and ; 1058213 contain the supplementary crystallographic data for this paper.† In the single crystal structure of compound **1**, the unit cell includes a large region of disordered solvent molecules, which could not be modeled as discrete atomic sites. We employed PLATON/SQUEEZE to calculate the diffraction contribution of the solvent molecules and, thereby, to produce a set of solvent-free diffraction intensities. For this structure, the SQUEEZE calculations showed a total solvent accessible area volume of 3211 Å^3^ and the residual electron density amounted to 1220 electrons per unit cell, corresponding to nearly 4 molecules of chloroform and 12 molecules of toluene (about 1 chloroform and 3 toluene molecules per asymmetric unit).†

### Computational details

Complete-active-space self-consistent field (CASSCF) calculations on the complete structures of compounds **1** and **2** on the basis of the X-ray determined geometry have been carried out with the MOLCAS 7.8 program package.^21^ Calculations for compound **3** were performed on the basis of the simulated structure optimized by the CAM-B3LYP functional, with a mixed basis set including SDDALL for the dysprosium ion and 6-311G(2df) for the other atoms, in the Gaussian 09 D.01 program.^27^ For the CASSCF calculations, the basis sets for all atoms were atomic natural orbitals from the MOLCAS ANO-RCC library: ANO-RCC-VTZP for Dy^III^ ions, VTZ for close O, S and N, and VDZ for distant atoms. The calculations employed the second-order Douglas–Kroll–Hess Hamiltonian, where scalar relativistic contractions were taken into account in the basis set, and the spin–orbit coupling was handled separately in the restricted active-space state interaction (RASSI-SO) procedure. The active electrons in 7 active spaces include all f electrons (CAS(9 in 7)) in the CASSCF calculation. To exclude all doubt we calculated all the roots in the active space. We have mixed the maximum number of spin-free states which was possible with our hardware (all from 21 sextets, 128 from 224 quadruplets and 130 from 490 doublets).
