# Supplementary material for: Rational enhancement of the energy barrier of bis(tetrapyrrole) dysprosium SMMs via replacing atom of porphyrin core
Source: Chem Sci. 2015 Jul 20;6(10):5947–54. doi: 10.1039/c5sc02314a (PMC5523118; doi:10.1039/c5sc02314a)
Supplement: Supplementary file 1 [file SC-006-C5SC02314A-s001.pdf]

Electronic Supplementary Material (ESI) for Chemical Science.
This journal is © The Royal Society of Chemistry 2015

Electronic Supplementary Information

Rational Enhancement of the Energy Barrier of Bis(tetrapyrrole) Dysprosium SMMs *via* Replacing Atom of Porphyrin Core

Wei Cao,[a] Chen Gao,[c] Yi-Quan Zhang,[d] Dongdong Qi,[a] Tao Liu,*[b] Chunying Duan,[b] Kang Wang,[a] Song Gao,*[c] and Jianzhuang Jiang*[a]

General procedure for preparing 2,5-bis[(4-tert-butyl)phenyl(hydroxy)- methyl]-furan and 2,5-bis[(4-tert-butyl)phenyl(hydroxy)-methyl]-thiophene: Both 2,5-bis[(4-tert-butyl)phenyl(hydroxy)-methyl]-furan and 2,5-bis[(4-tert-butyl)phenyl(hydroxy)-methyl]-thiophene were prepared according to the published procedure. Dry and freshly distilled *n*-hexane (50 mL) was added to a three-necked, round-bottomed flask (250 mL) that was flushed with dry nitrogen gas for 10 min. N,N,N`,N`-Tetramethylethylenediamine (TMEDA, 4.8 mL, 32 mmol) and *n*-BuLi (14 mL, 2.5 M in *n*-hexane, 35 mmol) were added and the solution was stirred under a dry nitrogen atmosphere for 10 min. Then, furan or thiophene (16 mmol) was added and the solution was gently heated at reflux for 1 h. The reaction mixture was then cooled to 0℃ in an ice bath. To which an ice cold solution of the 4-*tert*-butylbenzaldehyde (5.35 g, 33 mmol) in dry ether (32 mL) was slowly added. The reaction mixture was further stirred for 30 min and allowed to warm to room temperature, which was quenched by adding a saturated solution of NH_4Cl. The organic layer was extracted and dried with anhydrous Na_2SO_4. The solvent was removed on a rotary evaporator under reduced pressure to afford the crude compound, which was then purified by column chromatography on silica gel using ethyl acetate/petroleum ether (1:6), affording to the target compound as a white solid.

2,5-Bis[(4-tert-butyl)phenyl(hydroxy)-methyl]-furan: 2.77 g, 44 %; ^{1}H NMR (400 MHz, $CDCl_3$): δ=7.34-7.40 (m, 8H), 6.01 (d, 2H), 5.78 (s, 2H), 2.40 (s, 2H), 1.32 ppm (s, 18H).

2,5-Bis[(4-tert-butyl)phenyl(hydroxy)-methyl]-thiophene: 3.60 g, 55 %; ^{1}H NMR (400 MHz, $CDCl_3$): δ=7.34-7.40 (m, 8H), 6.69 (d, 2H), 5.92 (s, 2H), 2.57 (d, 2H), 1.33 ppm (s, 18H).

Preparation of 5,10,15,20-(4-tert-butyl)phenyl-21-oxaporphyrin: A solution of 4-tert-butylbenzaldehyde (0.97 g, 6 mmol), pyrrole (600 μL, 9 mmol), and 2,5-bis[(4-tert-butyl)phenyl(hydroxy)-methyl]-furan (1.18 g, 3 mmol) in dry dichloromethane (1 L) was degassed and purged with nitrogen. To start the condensation reaction, BF_3 diethyl etherate (40 μL, 0.3 mmol) was added, and the reaction mixture was stirred for 2 h in the dark under nitrogen atmosphere at room temperature. After addition of 2,3-dichloro-5,6-dicyano-1,4-benzoquinone (DDQ) (1.81 g, 8 mmol) and stirring on air for 10 h, the solvent was removed under reduced pressure. The product was then purified by column chromatography on basic aluminum oxide (dichloromethane) and recrystallized from *n*-hexane/dichloromethane, giving 5,10,15,20-(4-tert-butyl)phenyl-21-oxaporphyrin as a purple powder (300 mg, 12%). ^{1}H NMR (400 MHz, $CDCl_3$): δ=9.20 (s, 2H, *β*-furan-H), 8.92 (s, 2H, *β*-pyrrole-H), 8.56-8.63 (dd, 4H, *β*-pyrrole-H), 8.12-8.14 (dd, 8H, *o*-ArH), 7.75-7.77 (dd, 8H, *m*-Ar-H), 1.61 (s, 36H, t-butyl), -1.51 ppm (v br s, 1H, NH); UV-vis ($CHCl_3$) λ_{max} (logε): 424 (5.48), 510 (4.38), 544 (3.85), 612 (3.66), 674 (3.74) nm; MALDI-MS *m/z* 841.0, calcd. mass 840.5(M + H^+) .

Preparation of 5,10,15,20-(4-Tert-butyl)phenyl-21-thiaporphyrin: By employing the reaction for the preparation of 5,10,15,20-(4-tert-butyl)phenyl-21-oxaporphyrin with 4-tert-butylbenzaldehyde (2.91 g, 18 mmol), pyrrole (1400 μL, 21 mmol), and in particular 2,5-bis[(4-tert-butyl)phenyl(hydroxy)-methyl]-thiophene (1.18 g, 3 mmol) instead of 2,5-bis[(4-tert-butyl)phenyl(hydroxy)-methyl]-furan in dry dichloromethane (500 ml), the target core-modified porphyrin 5,10,15,20-(4-tert-butyl)phenyl-21-thiaporphyrin was isolated by chromatography on a silica gel column with chloroform and *n*-hexane (1:2 v/v) as the eluent followed by recrystallization from $CHCl_3$ and MeOH as a purple powder (385 mg, 15%). ^{1}H NMR (400 MHz, $CDCl_3$): δ=9.79 (s, 2H, *β*-thiophene-H), 8.94 (s, 2H, *β*-pyrrole-H), 8.64-8.73 (dd, 4H, *β*-pyrrole-H), 8.12-8.21 (dd, 8H, *o*-ArH), 7.74-7.84 (dd, 8H, *m*-Ar-H), 1.61 (s, 36H, t-butyl), -2.64 ppm (s, 1H, NH); UV-vis ($CHCl_3$) λ_{max} (logε): 432 (5.58), 516 (4.42), 552 (4.06), 618 (3.63), 678 (3.94) nm; MALDI-MS *m/z* 857.8, calcd. mass 856.4(M + H^+) .

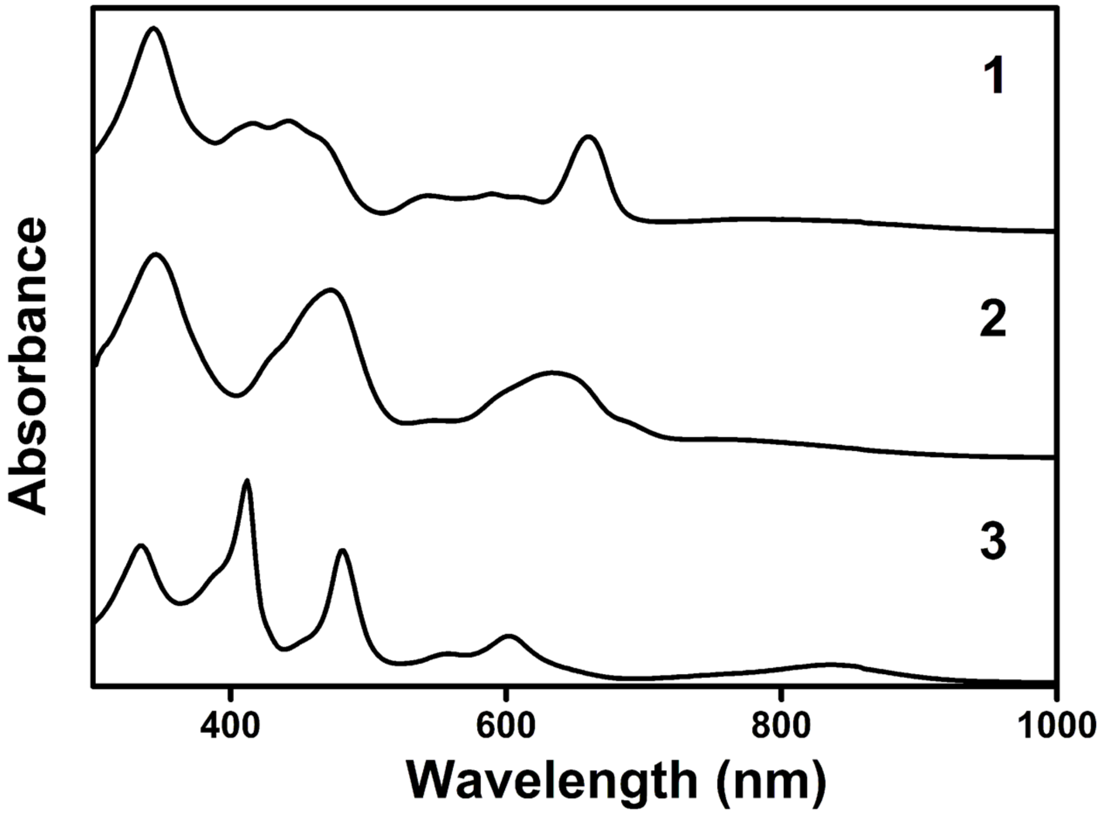

Figure S1. Electronic absorption spectra of the double-deckers **1-3** in $CHCl_3$.

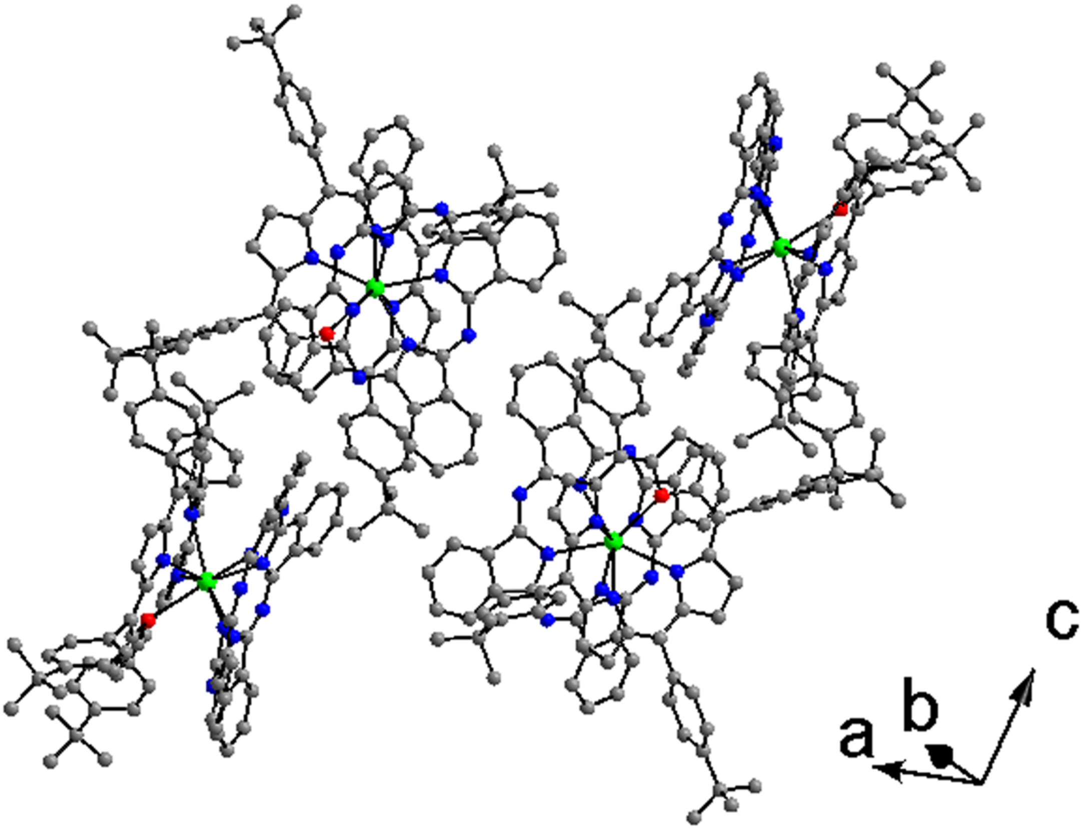

Figure S2. Molecular packing in single crystals of **1** with hydrogen atoms omitted for clarity.

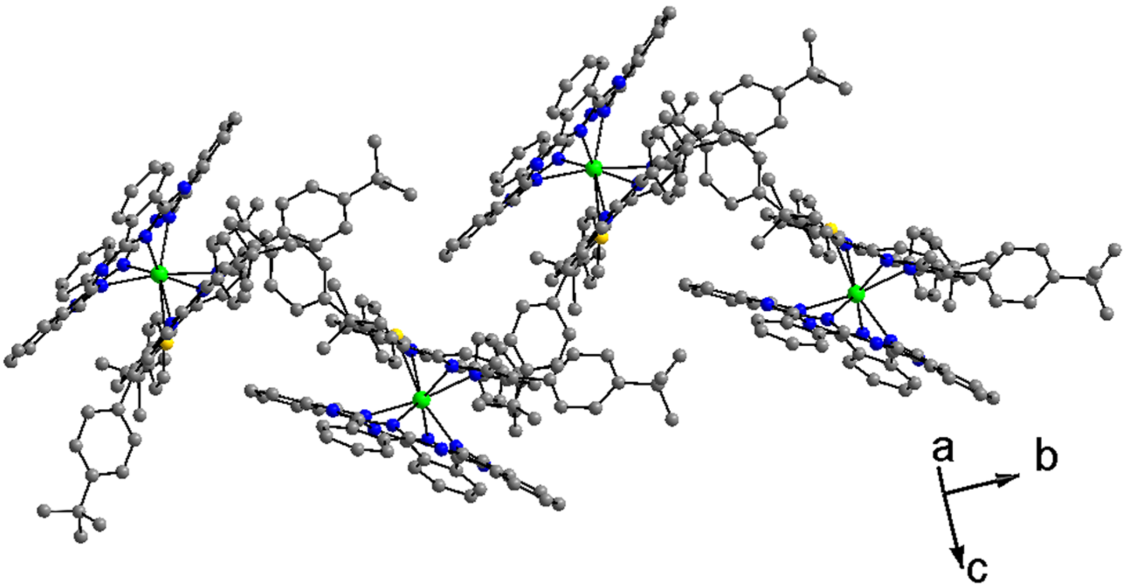

Figure S3. Molecular packing in single crystals of **2** with hydrogen atoms and solvent molecules omitted for clarity.

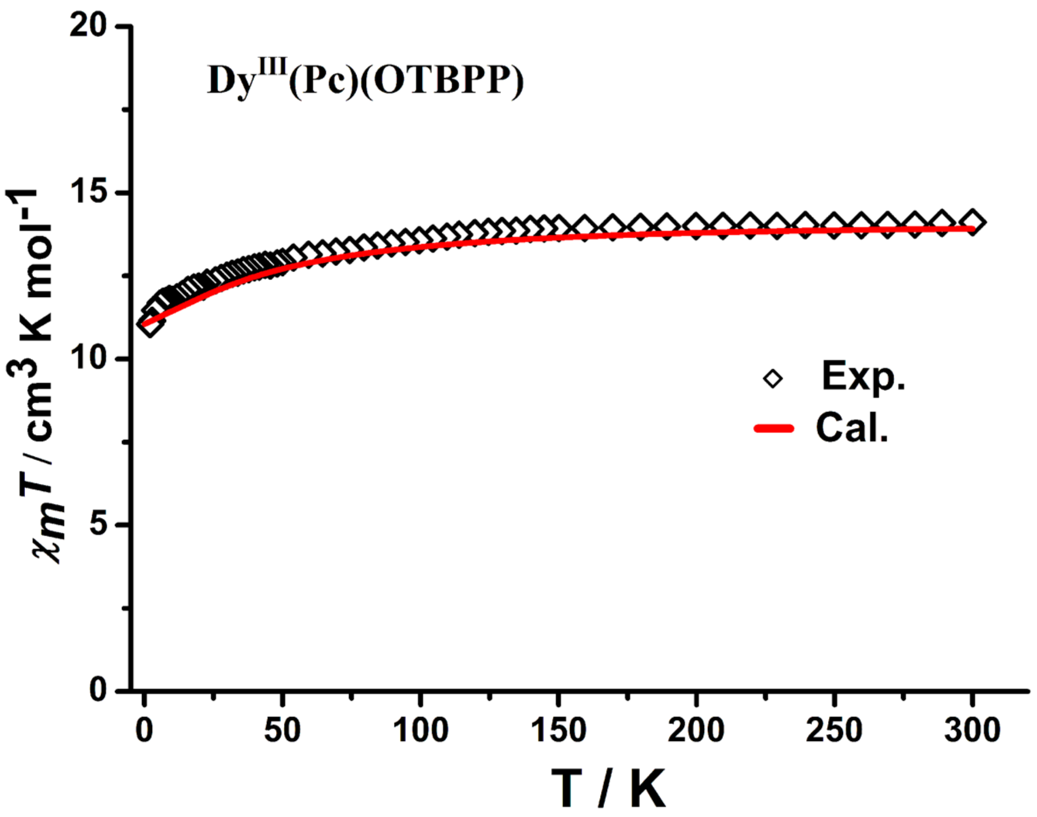

Figure S4. Temperature dependence of $\chi_m T$ for **1** by experimental measurement and theoretical calculation.

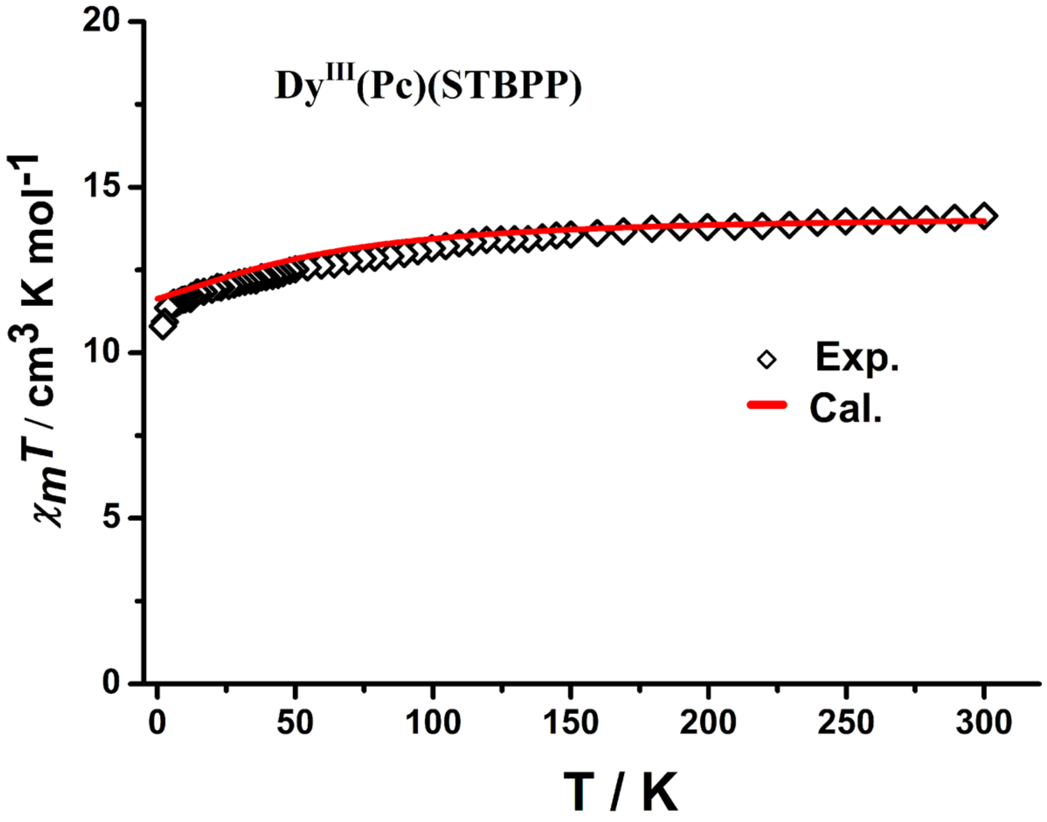

Figure S5. Temperature dependence of $\chi_m T$ for **2** by experimental measurement and theoretical calculation.

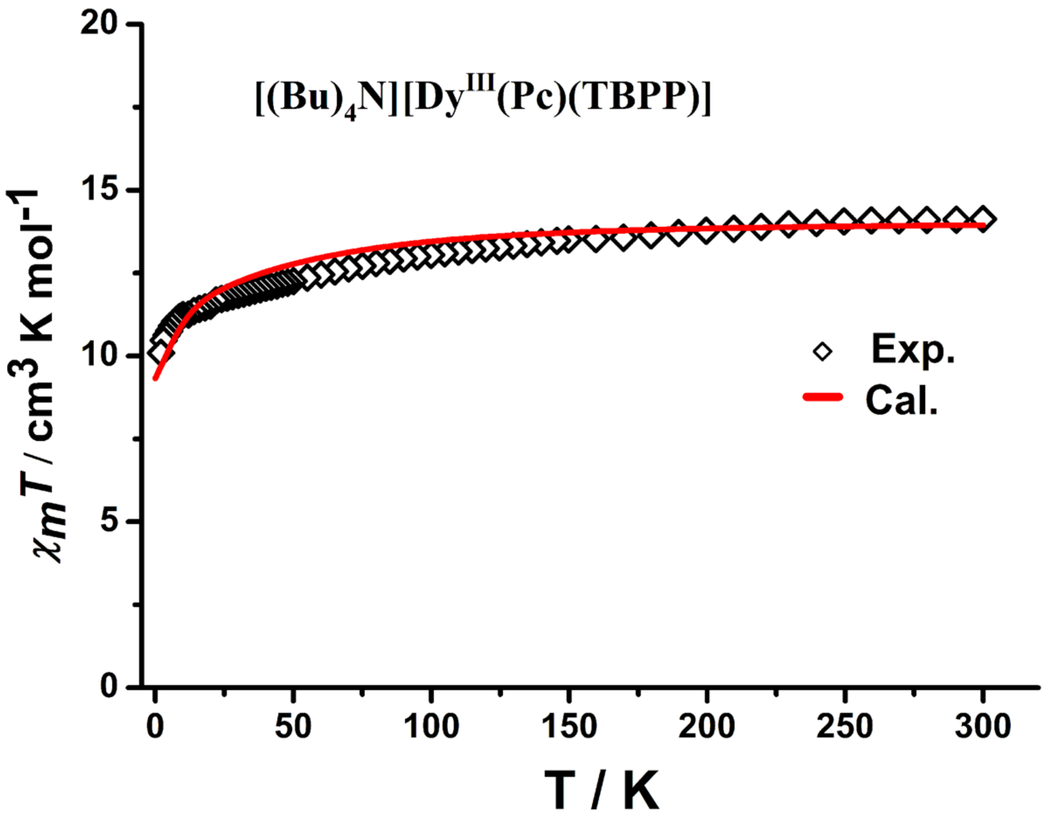

Figure S6. Temperature dependence of $\chi_m T$ for **3** by experimental measurement and theoretical calculation.

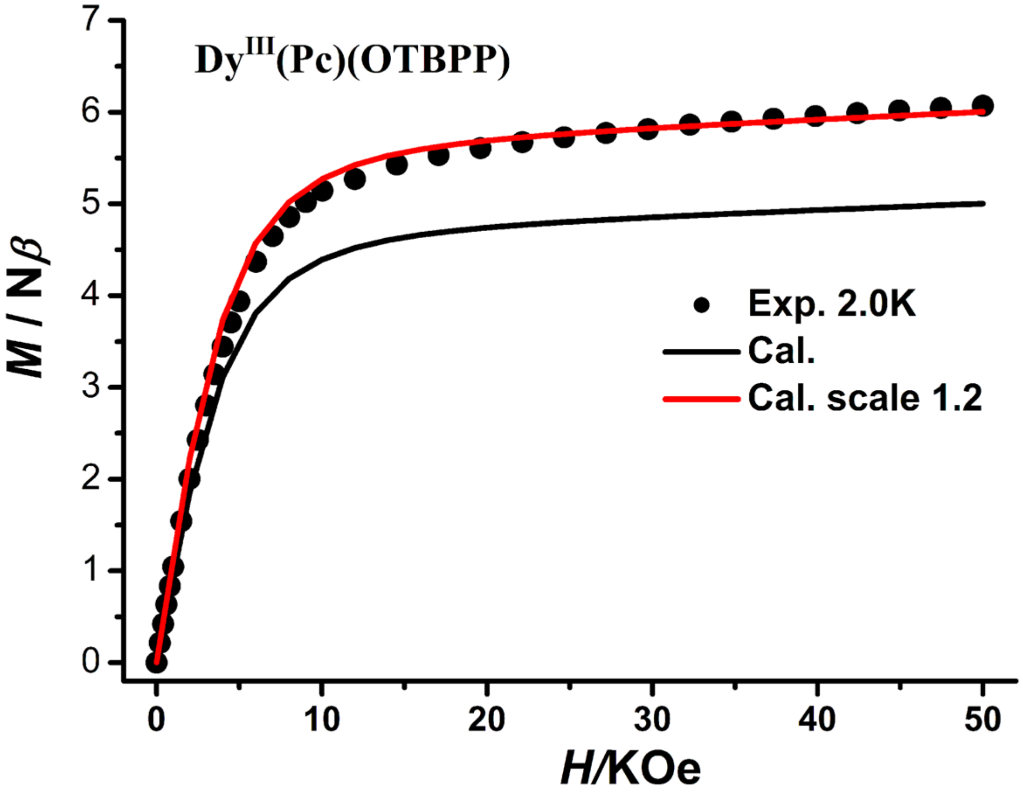

Figure S7. The *M vs H* curves for **1** at 2.0 K.

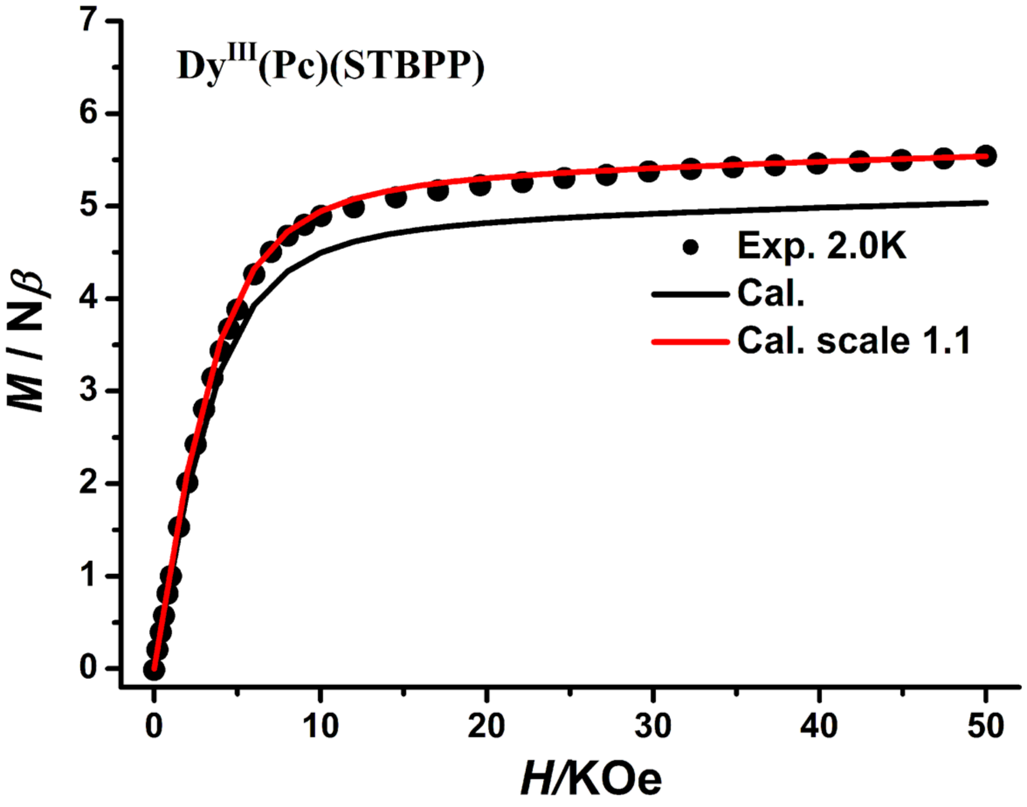

Figure S8. The *M vs H* curves for **2** at 2.0 K.

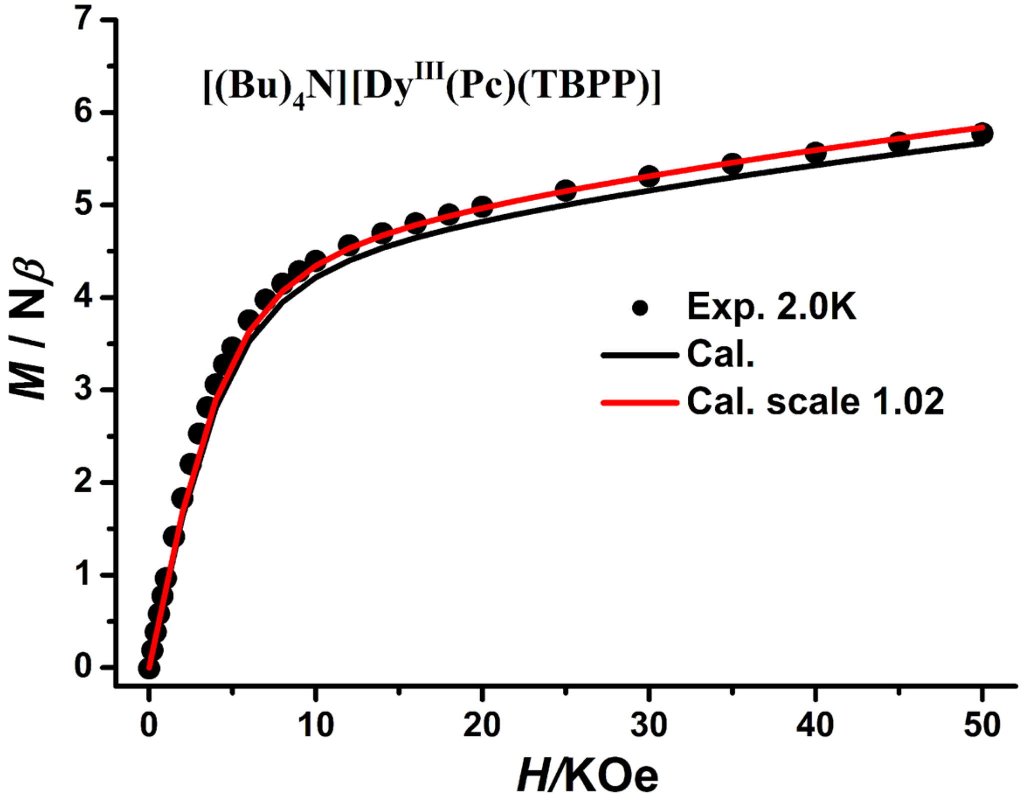

Figure S9. The *M vs H* curves for **3** at 2.0 K.

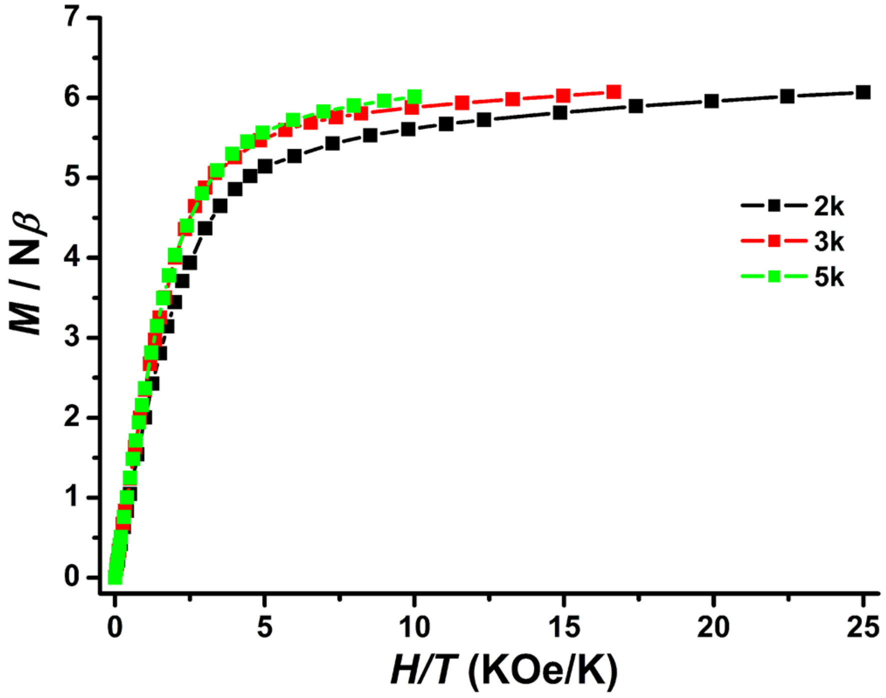

Figure S10. The *M vs H*/T curves for **1** at 2.0 K, 3.0 K and 5.0 K.

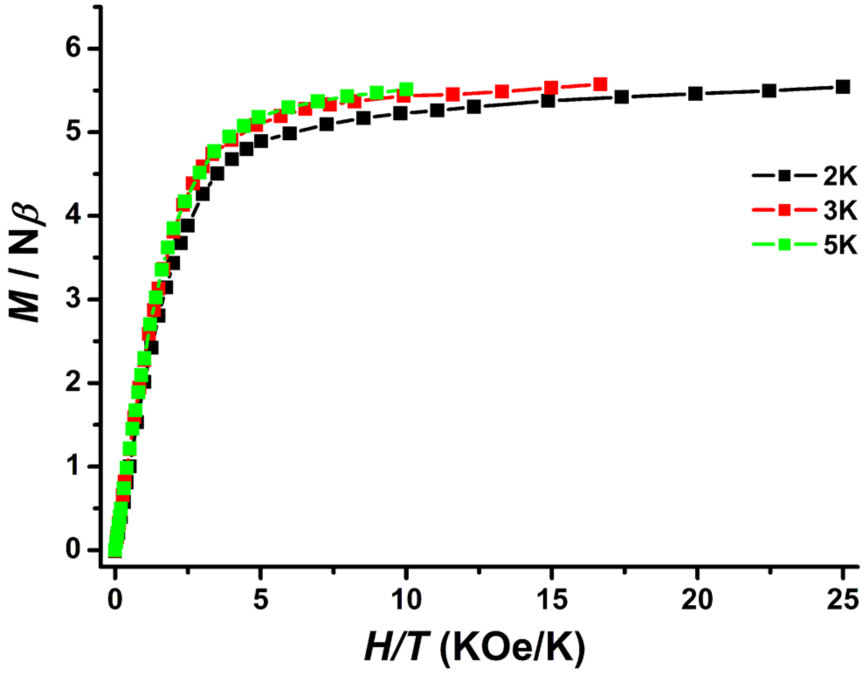

Figure S11. The *M vs H*/T curves for **2** at 2.0 K, 3.0 K and 5.0 K.

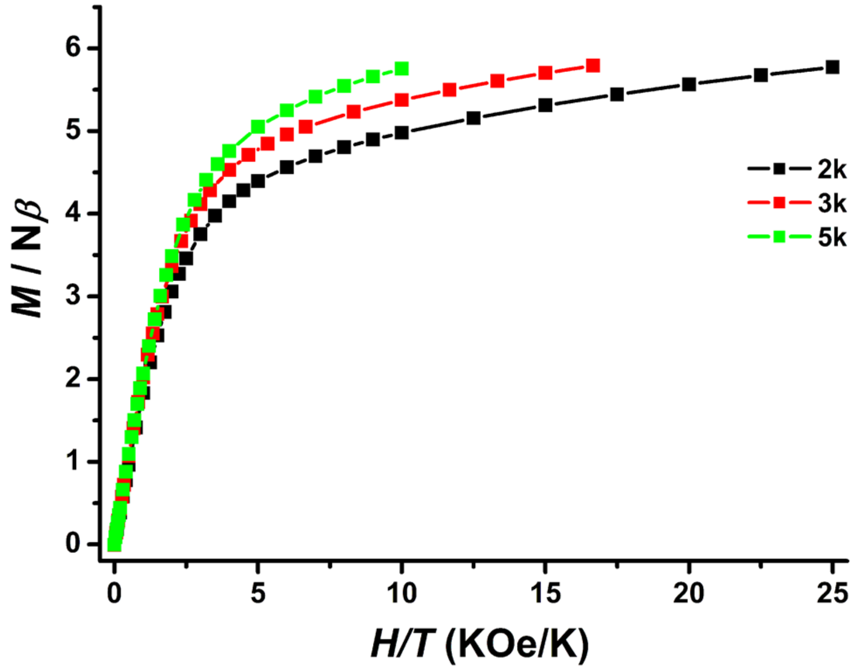

Figure S12. The *M vs H*/T curves for **3** at 2.0 K, 3.0 K and 5.0 K.

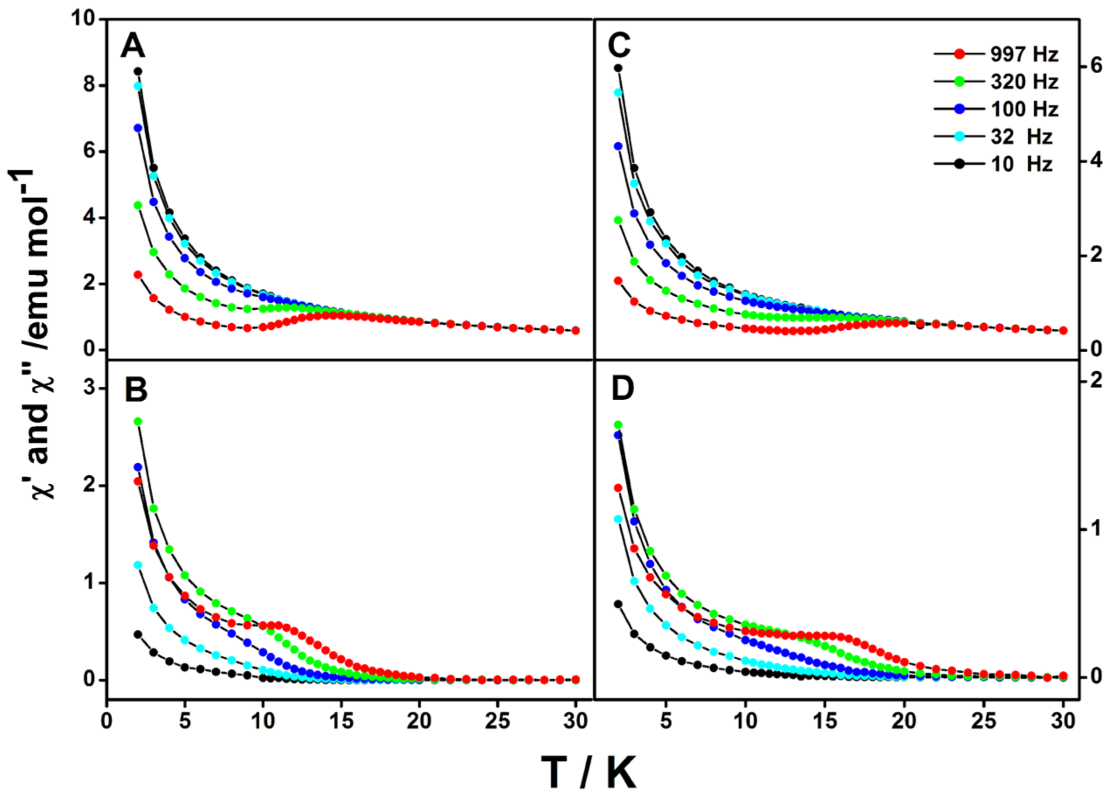

Figure S13. Temperature depenpence of the in-phase (χ') and out-of-phase (χ'') ac susceptibility of **1** (A, B) and **2** (C, D), respectively, under zero applied dc field.

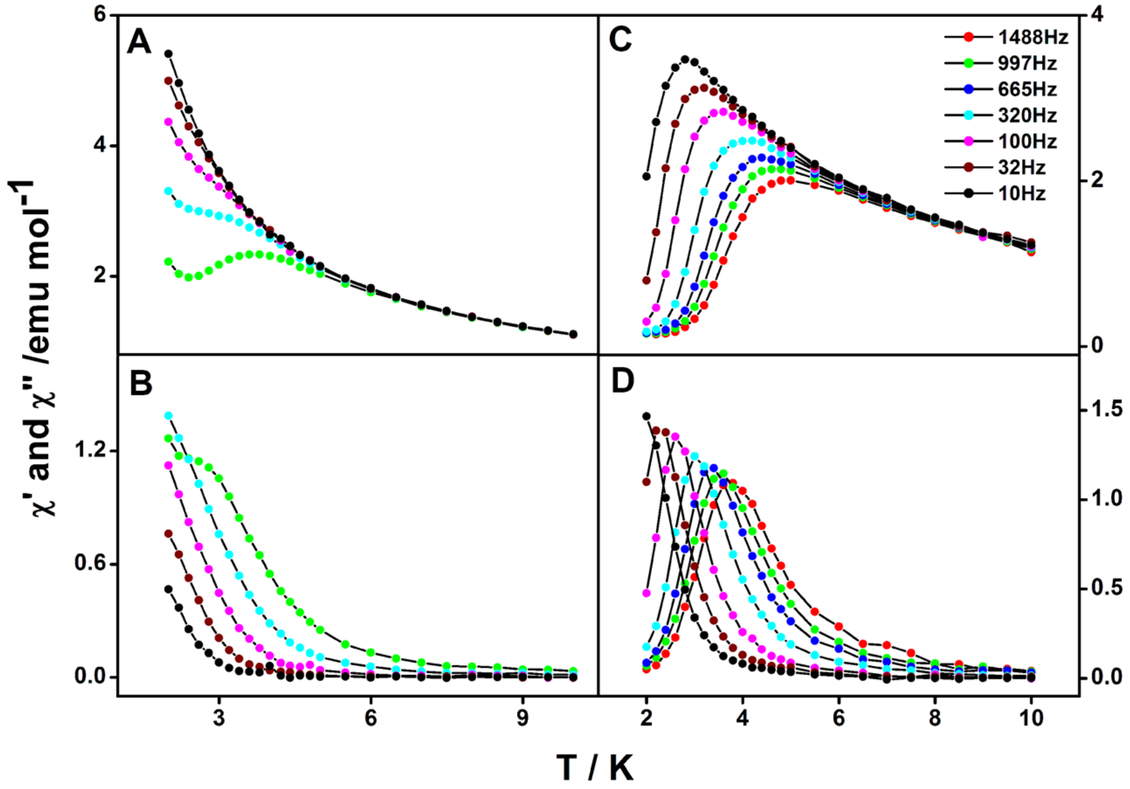

Figure S14. Temperature depenpence of the in-phase (χ') and out-of-phase (χ'') ac susceptibility of **3** under zero (A, B) and 2000Oe (C, D) applied dc field, respectively.

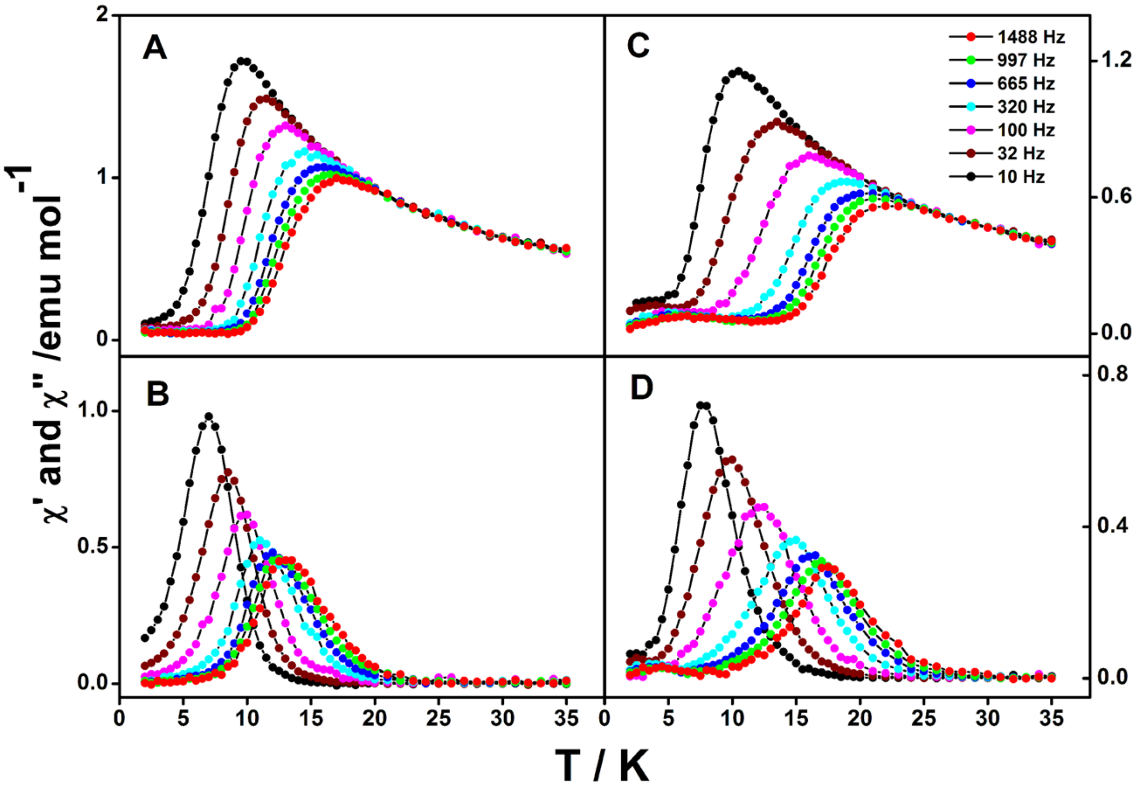

Figure S15. Temperature depenpence of the in-phase (χ') and out-of-phase (χ'') ac susceptibility of **1** (A, B) and **2** (C, D), respectively, under 2000Oe applied dc field.

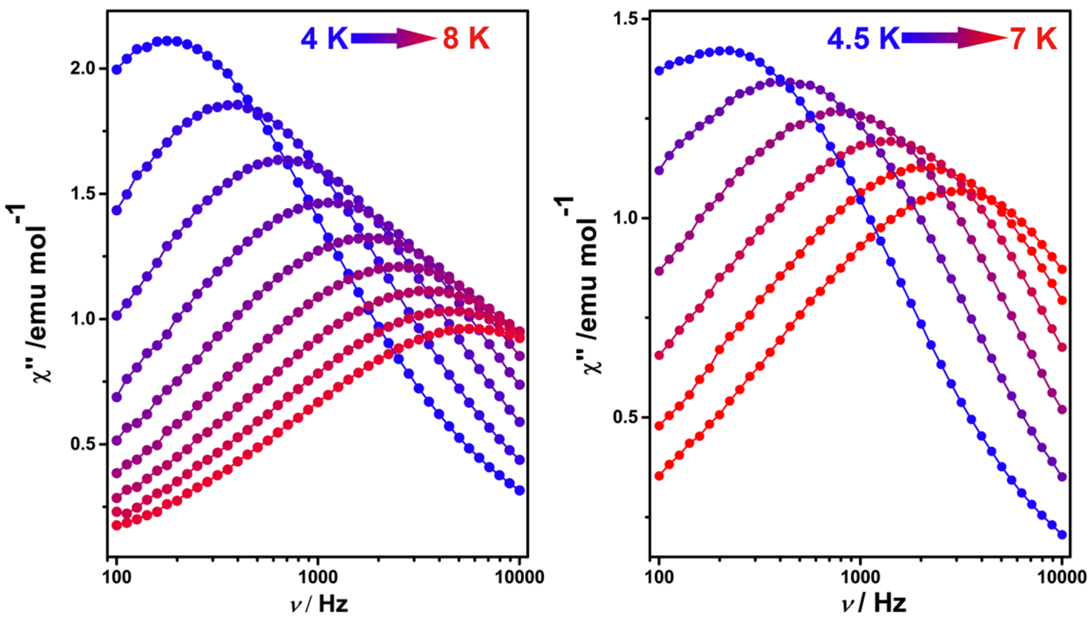

Figure S16. Frequency dependence of out of phase ac susceptibility signal of **3** without static field (left) or under a 2000 Oe field (right).

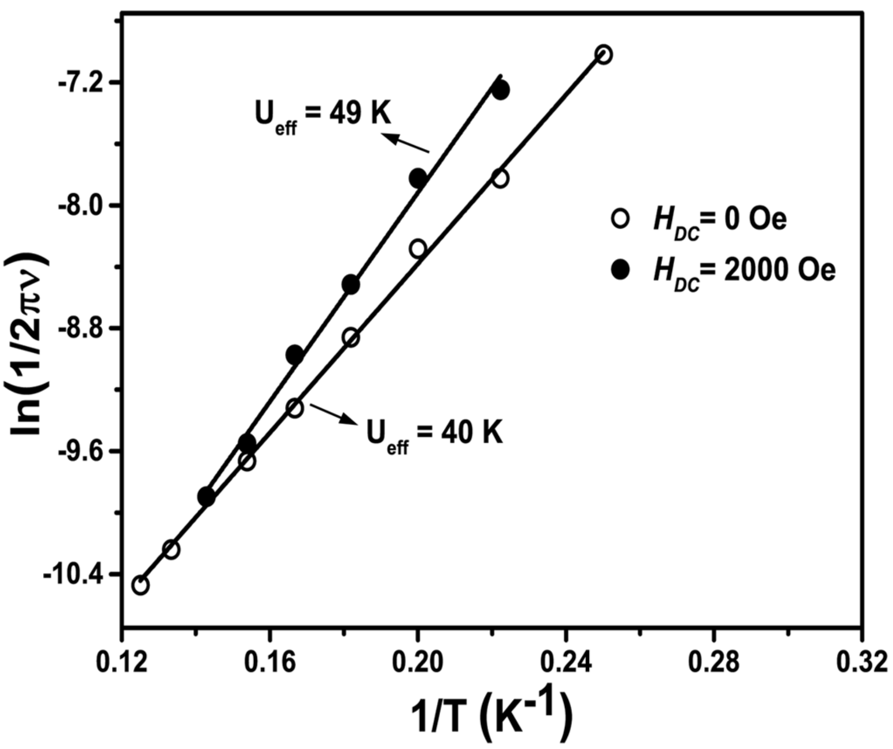

Figure S17. The plot of ln(τ) vs. 1/T for **3**.

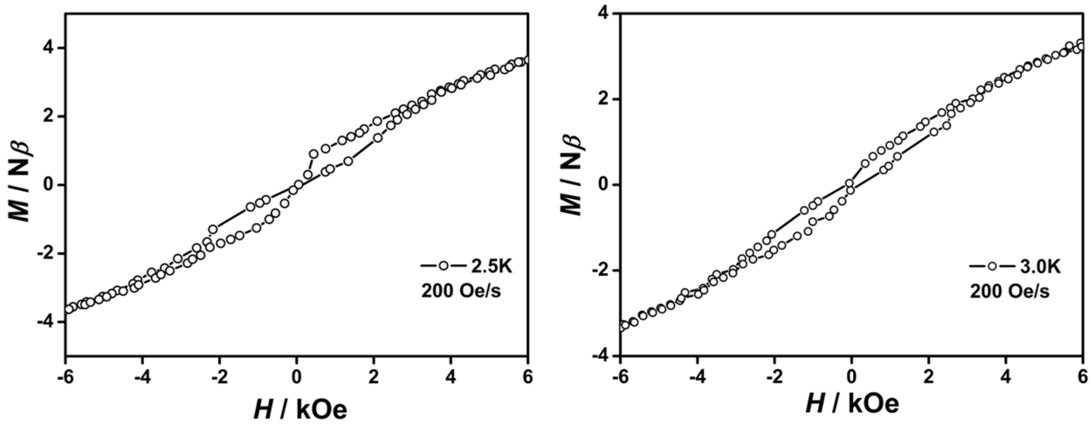

Figure S18. Hysteresis loop of **1** 2.5K and 3.0K with a 200 Oe/s sweep rate of the magnetic field.

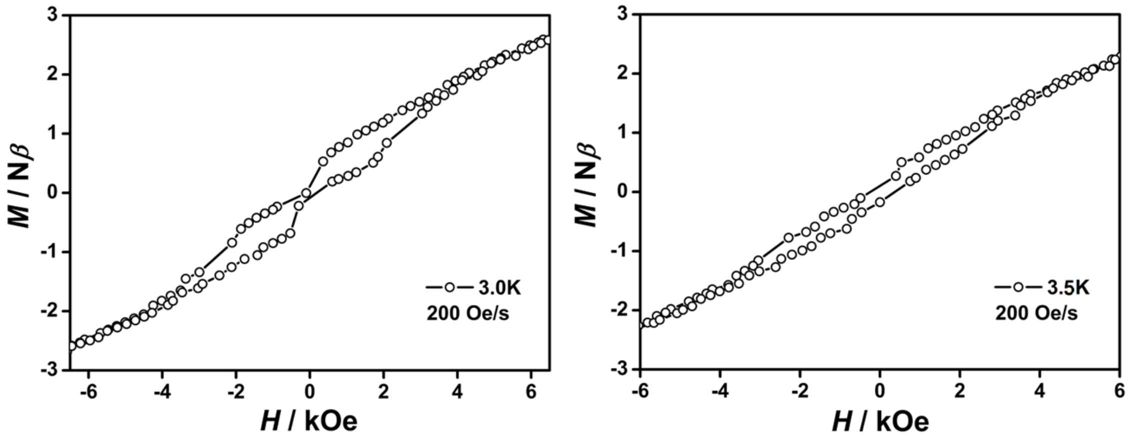

Figure S19. Hysteresis loop of **2** 3.0K and 3.5K with a 200 Oe/s sweep rate of the magnetic field.

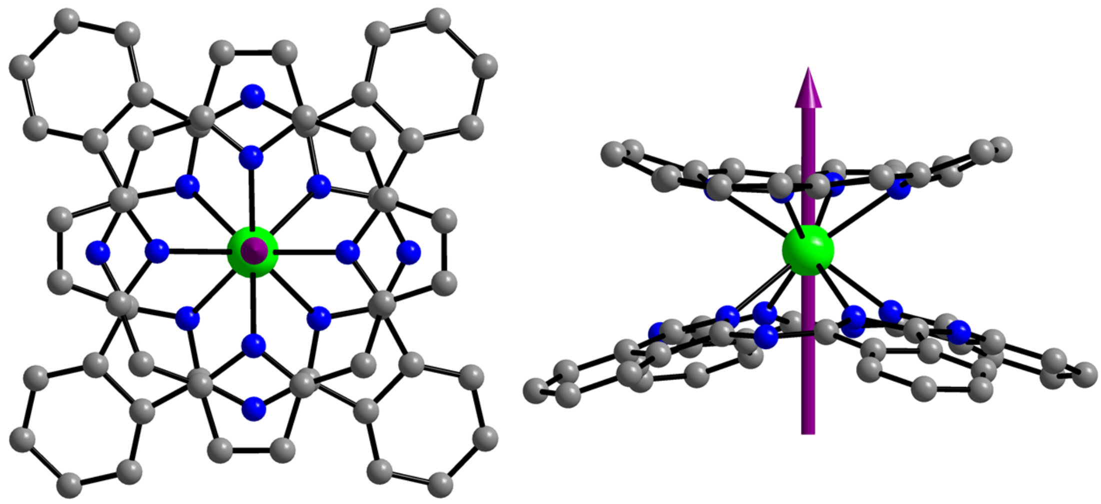

Figure S20. Simulated structure of **3** in top and side views with the hydrogen atoms, tetrabutyl ammonium ion and (4-*tert*-butyl)phenyl groups omitted for clarity. [Dy(III) green, C grey and N blue]. The purple arrows represent the easy axis calculated by using CASSCF calculation.

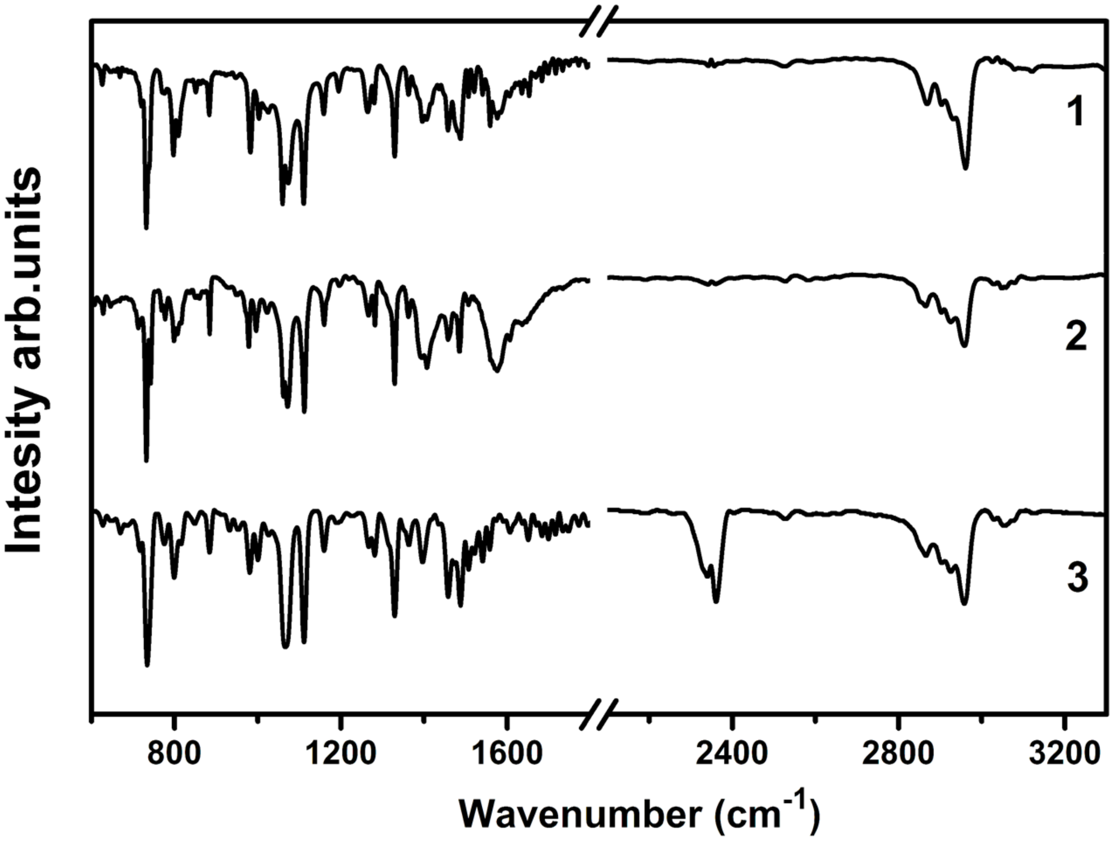

Figure S21. IR spectra of **1-3** in the region of 600-1800 and 2100-3300 cm^{-1}.

Table S1. Analytical and mass spectroscopic data for the double-deckers **1**-**3**.

Compound	Yield (%)	$[M+H]^+$ (*m/z*)[a,b]	Analysis (%)[a]			
			C	H	N	S
Dy^{III}(Pc)(OTBPP) (**1**)[c]	17	1514.5 (1514.6)	72.05(72.12)	4.99(5.13)	9.92(10.05)	------
Dy^{III}(Pc)(STBPP) (**2**)	3.1	1532.6 (1532.5)	72.31(72.21)	5.10(5.01)	9.95(10.07)	2.02(2.10)
$[(Bu)_4N][Dy^{III}(Pc)(TBPP)]$ (**3**)[d]	90	------ [e]	72.61 (72.44)	6.41 (6.53)	10.22 (10.17)	------

[a] Calculated values given in parentheses. [b] By MALDI-TOF mass spectrometry. The value corresponds to the most abundant isotopic peak of the protonated molecular ion $[M+H]^+$. [c] Contain 1 equiv. of solvated H_2O. [d] Contain 2 equiv. of solvated H_2O. [e] Only $[Dy^{III}(Pc)(TBPP)]^-$ ion was observed by MALDI-TOF mass spectrometry.

Table S2. Electronic absorption data for the double-deckers **1-3** in $CHCl_3$.

Compound	λ_{max}/nm (log ε)						
**1**	344(4.98)	416(4.73)	442 (4.74)	544 (4.23)	590 (4.26)	660(4.65)	780(3.83)
**2**	346(4.83)	472(4.74)	634(4.47)	--	--	--	--
**3**	334(4.99)	412(5.16)	482(4.98)	558(4.34)	602(4.54)	834(4.14)	--

Table S3. Structural data for the mixed double-deckers **1** and **2**.

	**1**	**2**
average Dy-N(pyrrole) bond distance [Å]	2.505	2.577
average Dy-N(isoindole) bond distance [Å]	2.413	2.376
Dy-N_3(Por) plane distance [Å]	1.405	1.641
Dy-N_4(Pc) plane distance [Å]	1.373	1.303
interplanar distance [Å]	2.778	2.944
dihedral angle between the N_3 and N_4 planes [°]	0.74	2.13
average dihedral angle ϕ for the Por ring [°] [a]	11.82	11.60
average dihedral angle ϕ for the Pc ring [°] [a]	15.07	12.96
average twist angle [°] [b]	44.22	43.30
the nearest Dy…Dy distance	12.481	9.311

[a] The average dihedral angle of the individual pyrrole or isoindole ring with respect to the corresponding N_3 or N_4 mean plane.

[b] Defined as the rotation angle of one macrocycle away from the eclipsed conformation of the two macrocycles.

Table S4. Selected bond lengths [Å] and angles [°] for complexes **1-3**.

	**1**	**2**	**3**[a]
Dy-X1 (X = O, S)	2.510(6)	2.769(2)	-----
Dy-N1	2.528(7)	2.683(5)	2.471
Dy-N2	2.494(6)	2.466(6)	2.451
Dy-N3	2.493(6)	2.582(5)	2.471
Dy-N4	2.428(7)	2.386(6)	2.451
Dy-N5	2.411(7)	2.364(5)	2.472
Dy-N6	2.390(7)	2.382(5)	2.462
Dy-N7	2.424(7)	2.371(5)	2.472
Dy-N8	-----	-----	2.462
X1-Dy1-N2 (X = O, S)	111.5(2)	92.59(15)	-----
N1-Dy1-N3	109.2(2)	102.25(19)	113.68
N2-Dy1-N4	-----	-----	112.95
N4-Dy1-N6	111.5(2)	112.90(18)	-----
N5-Dy1-N7	109.9(2)	114.01(18)	109.80
N6-Dy1-N8	-----	-----	110.18

[a] Selected bond lengths [Å] and angles [°] for complexes **3** was obtained on the basis of simulated structure.

Table S5. Crystallographic data for the mixed double-deckers **1** and **2**.

	**1**[a]	**2**·2.5CHCl$_3$
Molecular formula	$C_{92}H_{76}N_{11}ODy$	$C_{94.5}H_{78.5}N_{11}SCl_{7.5}Dy$
M	1514.14	1828.62
Crystal system	**Monoclinic**	**Monoclinic**
Space group	$P2_1/c$	$C2/c$
a/Å	17.5705(12)	31.8037(11)
b/Å	22.6256(12)	17.2640(4)
c/Å	24.6091(11)	32.0054(8)
α/°	90	90
β/°	100.073(6)	102.045(3)
γ/°	90	90
U/Å^3	9632.4(9)	17186.0(8)
Z	4	8
F(000)	3116	7456
D_c/Mg m^{-3}	1.044	1.413
μ/mm^{-1}	4.494	1.181
Data collection range/°	3.22 to 62.99	3.05 to 25.00
Reflections measured	29858	31275
Independent reflections	15393 (Rint = 0.0861)	15106 (Rint = 0.0404)
Parameters	926	1029
R_1 [I>2$\sigma(I)$]	0.0992	0.0684
wR_2 [I>2$\sigma(I)$]	0.2516	0.1745
Goodness of fit	0.939	1.038

[a] One chloroform and three toluene molecules were removed by PLATON/SQUEEZE.

Table S6. Calculated energy levels (cm^{-1}), m_J values and g tensors (x, y, z) of the lowest Kramers doublets (KDs) for complexes **1-3**.

KD	**1**					**2**					**3**				
	***E*/cm^-1^**	m_J	g_x	g_y	g_z	***E*/cm^-1^**	m_J	g_x	g_y	g_z	***E*/cm^-1^**	m_J	g_x	g_y	g_z
1	0.00	±15/2	0.002	0.005	18.800	0.00	±15/2	0.008	0.014	19.285	0.00	±13/2	0.006	0.007	17.270
2	96.33	±13/2	0.015	0.033	15.309	120.51	±13/2	0.093	0.151	15.735	26.30	±11/2	0.009	0.020	14.544
3	192.18	±11/2	0.290	0.302	13.080	204.94	±11/2	0.373	0.446	13.679	110.35	±9/2	0.081	0.108	11.822
4	275.46	±9/2	1.429	1.577	13.760	277.03	±9/2	0.491	0.643	16.337	169.48	±15/2	0.026	0.038	19.306
5	307.93	±5/2	2.363	2.859	12.678	312.70	±5/2	3.361	5.708	9.136	195.18	±7/2	0.772	0.891	9.642
6	368.24	±3/2	7.495	4.850	0.059	377.93	±1/2	3.074	3.345	12.979	253.64	±5/2	1.128	3.006	6.299
7	412.68	±1/2	1.693	6.279	13.092	447.05	±3/2	0.638	1.189	18.779	291.69	±3/2	9.265	7.755	2.699
8	571.62	±7/2	0.022	0.035	19.649	604.23	±7/2	0.008	0.041	19.680	359.57	±1/2	0.261	0.691	17.921

Table S7. Mulliken charge distribution of selected atoms for complexes **1-3**.

**Parameter**	**1**	**2**	**3**
Charge(X1) (X = O, S)	-0.75	0.15	-----
Charge(N1)	-0.89	-0.89	-0.84
Charge(N2)	-0.83	-0.86	-0.86
Charge(N3)	-0.86	-0.88	-0.84
Charge(N4)	-0.86	-0.84	-0.84
Charge(N5)	-0.85	-0.84	-0.83
Charge(N6)	-0.84	-0.86	-0.82
Charge(N7)	-0.81	-0.84	-0.82
Charge(N8)	-----	-----	-0.83

Table S8. Decomposition of the RASSI wave functions corresponding to the lowest atomic multiplet J = 15/2 in wave functions with definite projection of the total moment | JM > for compound **1**.

| JM >	wave function 1		wave function 2	
-15/2	0.863046	-0.223712	0.000000	0.000000
-13/2	-0.204147	0.009276	0.000040	-0.000149
-11/2	-0.376461	-0.012652	0.000014	0.000135
-9/2	-0.113365	-0.036675	0.000510	0.000071
-7/2	0.044563	0.021103	-0.001478	-0.000345
-5/2	0.048194	0.043660	-0.000243	0.000100
-3/2	0.015087	0.016163	0.002122	0.003245
-1/2	0.000006	-0.004939	-0.002006	-0.008285
+1/2	-0.000137	-0.008523	0.001245	0.004780
+3/2	0.001240	-0.003674	-0.010548	0.019431
+5/2	0.000260	0.000036	0.035697	-0.054357
+7/2	-0.001344	0.000705	-0.037843	0.031610
+9/2	-0.000476	0.000196	-0.100536	0.063948
+11/2	-0.000020	-0.000134	0.361242	-0.106709
+13/2	-0.000076	-0.000135	-0.199944	0.042245
+15/2	0.000000	0.000000	-0.891569	0.000000

Table S9. Decomposition of the RASSI wave functions corresponding to the lowest atomic multiplet J = 15/2 in wave functions with definite projection of the total moment | JM > for compound **2**.

| JM >	wave function 1		wave function 2	
-15/2	0.090011	0.937585	0.000000	0.000000
-13/2	-0.025699	0.118303	0.000373	-0.000456
-11/2	0.106970	-0.253362	0.000371	-0.000275
-9/2	-0.095706	0.099122	0.000382	0.000366
-7/2	0.025162	-0.005890	0.000272	0.000713
-5/2	0.040678	-0.017129	-0.002786	-0.001389
-3/2	-0.029136	0.001758	-0.003891	-0.003517
-1/2	0.005107	0.003854	0.000294	-0.002980
+1/2	0.002938	-0.000578	0.004325	0.004716
+3/2	-0.003873	-0.003538	0.001034	0.029170
+5/2	0.001649	0.002641	-0.013163	0.042129
+7/2	0.000735	0.000203	0.003459	-0.025609
+9/2	-0.000401	-0.000345	0.089523	-0.104741
+11/2	-0.000238	0.000395	0.241980	-0.130692
+13/2	0.000419	-0.000415	0.115305	-0.036887
+15/2	0.000000	0.000000	-0.941896	0.000000

Table S10. Decomposition of the RASSI wave functions corresponding to the lowest atomic multiplet J = 15/2 in wave functions with definite projection of the total moment | JM > for compound **3**.

| JM >	wave function 1		wave function 2	
-15/2	0.000000	0.000000	-0.027316	-0.022142
-13/2	-0.000452	0.000026	-0.395422	0.915690
-11/2	0.000198	-0.000331	-0.023300	-0.001798
-9/2	0.000032	0.000003	0.039988	-0.030978
-7/2	-0.000366	0.000270	-0.000925	0.001247
-5/2	-0.000006	0.000007	0.003301	0.028197
-3/2	0.000128	-0.000834	-0.001275	0.000372
-1/2	0.000028	-0.000040	-0.000773	0.000798
+1/2	0.000098	0.001107	-0.000003	0.000049
+3/2	-0.000756	-0.001091	0.000426	-0.000728
+5/2	-0.020320	0.019826	0.000000	-0.000009
+7/2	0.000067	-0.001552	0.000114	0.000440
+9/2	-0.011558	-0.049245	0.000027	0.000018
+11/2	-0.019233	-0.013275	0.000054	-0.000382
+13/2	-0.269424	0.960342	-0.000335	-0.000305
+15/2	-0.035163	0.000000	0.000000	0.000000
